# EZH2 crosstalk with RNA methylation promotes prostate cancer progression through modulation of m^6^A autoregulation pathway

**DOI:** 10.1172/JCI195840

**Published:** 2025-11-18

**Authors:** Yang Yi, Joshua Fry, Chaehyun Yum, Rui Wang, Siqi Wu, Sharath Narayan, Qi Liu, Xingxing Zhang, Htoo Zarni Oo, Ning Xie, Yanqiang Li, Xinlei Gao, Xufen Yu, Xiaoping Hu, Qiaqia Li, Kemal Keseroglu, Ertuğrul M. Özbudak, Sarki A. Abdulkadir, Kaifu Chen, Jian Jin, Jonathan C. Zhao, Xuesen Dong, Daniel Arango, Rendong Yang, Qi Cao

**Affiliations:** 1 Department of Urology and; 2 Robert H. Lurie Comprehensive Cancer Center, Feinberg School of Medicine, Northwestern University, Chicago, Illinois, USA.; 3 Bioinformatics and Computational Biology Program, University of Minnesota, Minneapolis, Minnesota, USA.; 4 Driskill Graduate Program in Life Sciences, Feinberg School of Medicine, Northwestern University, Chicago, Illinois, USA.; 5 Vancouver Prostate Centre, Vancouver General Hospital, Vancouver, British Columbia, Canada.; 6 Department of Urologic Sciences, University of British Columbia, Vancouver, British Columbia, Canada.; 7 Basic and Translational Research Division, Department of Cardiology, Boston Children’s Hospital, Boston, Massachusetts, USA.; 8 Department of Pediatrics, Harvard Medical School, Boston, Massachusetts, USA.; 9 Prostate Cancer Program, Dana-Farber Harvard Cancer Center, Boston, Massachusetts, USA.; 10 Mount Sinai Center for Therapeutics Discovery, Departments of Pharmacological Sciences and Oncological Sciences, Tisch Cancer Institute, Icahn School of Medicine at Mount Sinai, New York, New York, USA.; 11 Department of Cell and Developmental Biology and; 12 Department of Pathology, Feinberg School of Medicine, Northwestern University, Chicago, Illinois, USA.; 13 Department of Human Genetics and; 14 Winship Cancer Institute, Emory University School of Medicine, Atlanta, Georgia, USA.; 15 Department of Pharmacology, Feinberg School of Medicine, Northwestern University, Chicago, Illinois, USA.

**Keywords:** Cell biology, Oncology, Prostate cancer

## Abstract

N6-methyladenosine (m^6^A), the most predominant RNA modification in humans, participates in various fundamental and pathological bioprocesses. Dynamic manipulation of m^6^A deposition in the transcriptome is critical for cancer progression, though how this regulation is achieved remains understudied. Here, we report that, in prostate cancer (PCa), Polycomb group (PcG) protein Enhancer of Zeste Homolog 2 (EZH2) exerts an additional function in m^6^A regulation via its enzymatic activity. Mechanistically, EZH2 methylates and stabilizes FOXA1 proteins from degradation, which, in turn, facilitates the transcription of m^6^A reader YTHDF1. Through activating an m^6^A autoregulation pathway, YTHDF1 enhances the translation of METTL14 and WTAP, 2 critical components of the m^6^A methyltransferase complex (MTC), and thereby upregulates the global m^6^A level in PCa cells. We further demonstrate that inhibiting the catalytic activity of EZH2 suppresses the translation process globally through targeting the YTHDF1-m^6^A axis. By disrupting both the expression and interaction of key m^6^A MTC subunits, combinational treatment of EZH2 degrader MS8815 and m^6^A inhibitor STM2457 mitigates prostate tumor growth synergistically. Together, our study decodes a previously hidden interrelationship between EZH2 and mRNA modification, which may be leveraged to advance the EZH2-targeting curative strategies in cancer.

## Abstract 2

Our current article identifies EZH2 as a master regulator to reshape the global m^6^A methylome in prostate cancer cells and uncover the underlying mechanism.

## Graphical abstract

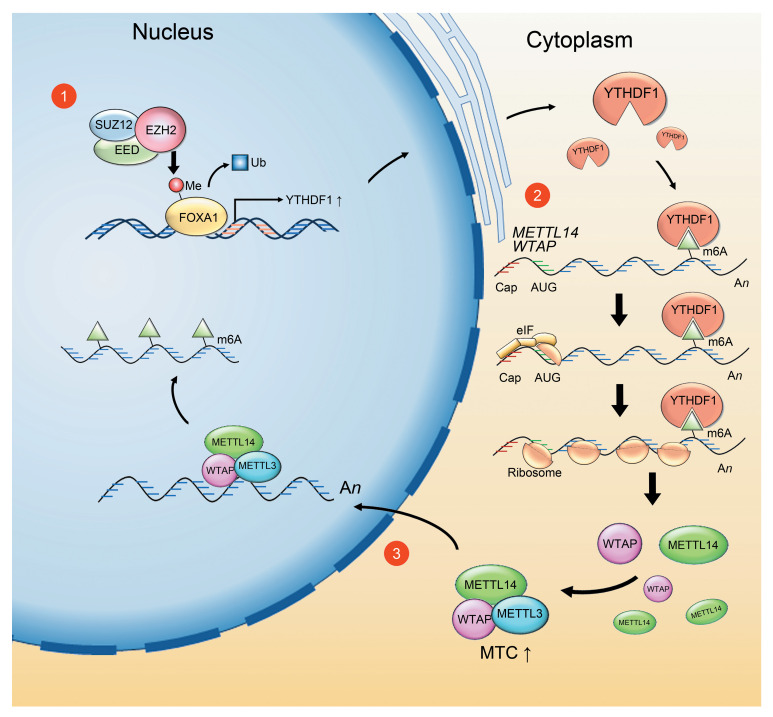

## Introduction

As the most prevalent modification type on mRNAs, N6-methyladenosine (m^6^A) plays a pivotal role in governing RNA functions and metabolism (1, 2). Aberrant m^6^A modifications are frequently observed in cancer cells and are associated with tumor progression, immune response, and drug resistance (3–5). Notably, a hyper-m^6^A state is a feature of prostate cancer (PCa), which may be induced by the upregulation of m^6^A methyltransferase complex members (“writers”) (6, 7) or downregulation of m^6^A demethylases (“erasers”) (8, 9). In addition, dysregulation of a number of m^6^A binding proteins (“readers”) are also characterized in PCa (10, 11), which, together, facilitate PCa tumorigenesis and metastasis. However, how these m^6^A regulators are dynamically manipulated to maintain a hyper-m^6^A state in PCa cells is yet to be discovered.

Enhancer of Zeste Homolog 2 (EZH2) is the catalytic subunit of polycomb repressive complex 2 (PRC2), which canonically mediates histone H3 lysine 27 trimethylation (H3K27me3) to maintain a repressive chromatin state (12, 13). Since the initial discovery of EZH2 overexpression in PCa, mounting studies, including ours, have demonstrated the multifaceted functions of EZH2 in promoting oncogenesis, which can be exerted via both lysine methylation–dependent and –independent mechanisms (14–17).

Emerging evidence has supported a notion that the sophisticated crosstalk between histone and m^6^A modifications is critical for the precise and synchronous regulation of gene expression (18). For instance, histone mark of H3K36me3 could be recognized and bound directly by m^6^A writer METTL14, which, in turn, facilitates the deposition of m^6^A to nascent RNAs (19). Another study revealed that m^6^A guides the demethylation of histone H3K9me2 cotranscriptionally to promote gene expression (20). In addition, histone H3K9-specific demethylase KDM4C was reported to control the expression of m^6^A eraser ALKBH5 to reshape the m^6^A pattern in cancer cells (21). With regard to the interplay between m^6^A and H3K27me3, previous investigations have largely focused on the presence of m^6^A on transcripts of *EZH2* or histone demethylase *KDM6B* and their influences on H3K27me3 level (5, 22–24), while whether EZH2 can remodel the global m^6^A landscape in the opposite regulatory direction via its regulation of lysine methylation remains elusive.

By combing the Nanopore direct RNA sequencing (Nanopore-seq) technique with various molecular approaches, our current article identifies EZH2 as a master regulator to reshape the global m^6^A methylome in PCa cells. We reveal that EZH2 maintains a hyper-m^6^A state in cancer cells by activating a YTHDF1-mediated m^6^A autoregulation pathway. On this basis, we further deconstruct how the EZH2-m^6^A crosstalk facilitates PCa tumorigenesis and unveil its clinical relevance.

## Results

### EZH2 promotes RNA m^6^A methylation in PCa cells.

**Figure 1:**
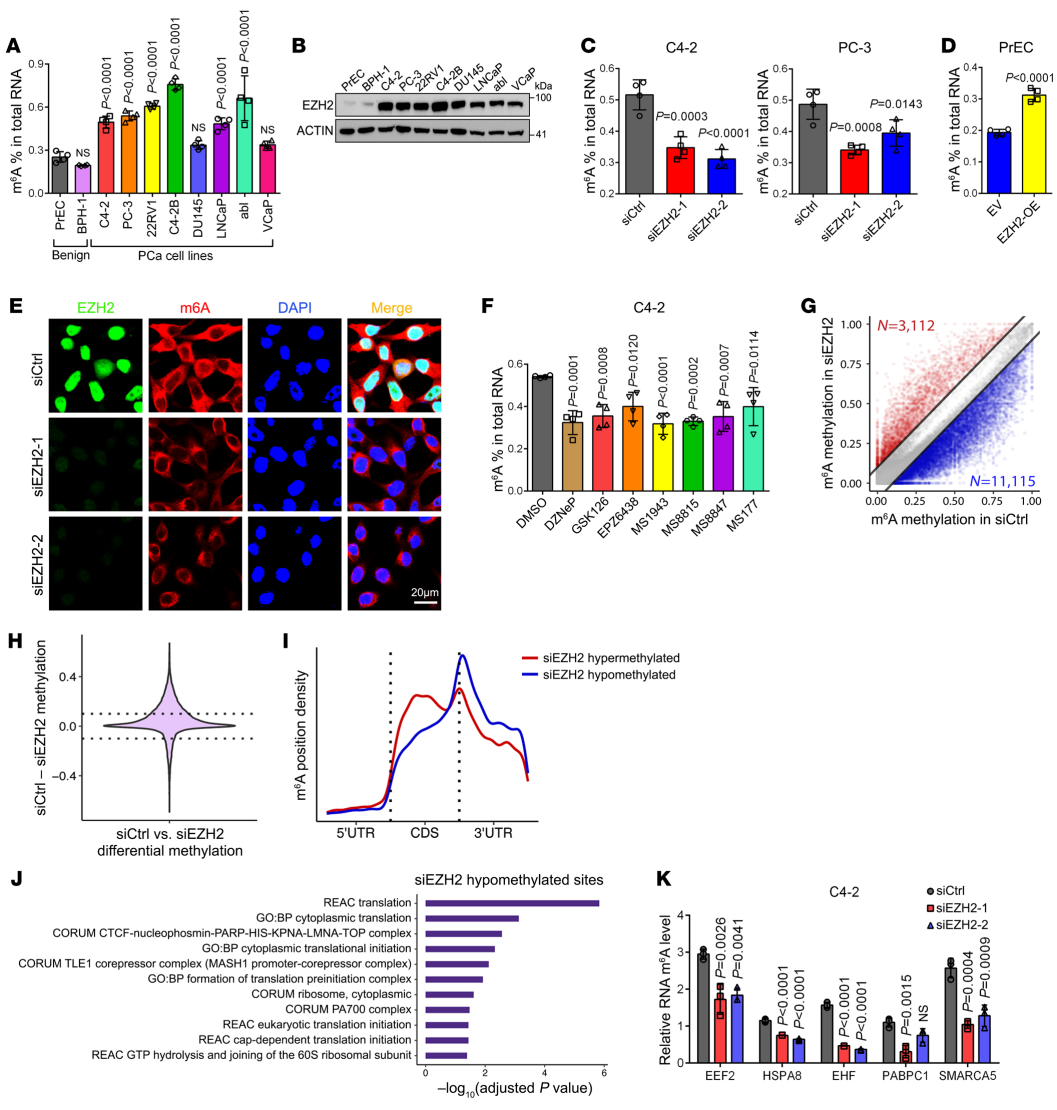
EZH2 maintains a hyper-m^6^A state in PCa cells. (**A**) The m^6^A ELISA to measure the global m^6^A levels in PrEC and BPH-1 benign prostate cells, along with a panel of PCa cell lines. (**B**) Western blot to detect the EZH2 expression level in all cell types tested in **A**. (**C** and **D**) The m^6^A ELISA to measure the global m^6^A levels in 2 PCa cell lines upon EZH2 knockdown (**C**), or in PrEC cells with EZH2 overexpression (**D**). (**E**) Representative fluorescence images to show the RNA m^6^A staining in control and EZH2-deficient C4-2 cells. Endogenous EZH2 were costained by anti-EZH2 antibody, and the nuclei were visualized by DAPI (Scale bar: 20 μm). (**F**) The m^6^A ELISA to measure the global m^6^A levels in C4-2 cells treated with a series of EZH2 inhibitors. For DZNeP, GSK126, and EPZ6438, a concentration of 5 μM was used. For all the MS drugs, a concentration of 1 μM was used. (**G**) Scatter plot showing the m^6^A methylation in C4-2 cells upon EZH2 depletion, with siEZH2 hypomethylated sites in blue and hypermethylated sites in red. (**H**) Violin plot to show the differential m^6^A ratio upon EZH2 knockdown in C4-2 cells. (**I**) The distribution of EZH2-affected m^6^A sites across the transcript. (**J**) Gene Ontology enrichment analysis of genes with hypomethylated m^6^A sites upon EZH2 knockdown in C4-2 cells. Statistical significance was assessed using the hypergeometric test with *P* values corrected for multiple testing with the g:SCS method using g:Profiler. (**K**) The m^6^A CUT&RUN-qPCR analysis to validate the EZH2-affected m^6^A sites in each indicated transcript of C4-2 cells. One-way ANOVA followed by Dunnett’s multiple-comparison test was used for statistical analysis in **A**, **C**, **F**, and **K**. Two-tailed Student’s *t* test was used in **D**.

To investigate the potential role of EZH2 in m^6^A regulation, we first measured the m^6^A levels in a panel of PCa cell lines along with human normal primary prostate epithelial cells (PrEC) and benign prostatic hyperplasia cell line BPH-1 using m^6^A ELISA. As compared with PrEC, which bears rare endogenous EZH2, most of the PCa cell lines characterized by EZH2 overexpression were accompanied with elevated m^6^A ratio (Figure 1, A and B). In addition, siRNA-mediated EZH2 knockdown in PCa cell lines of C4-2 and PC-3 significantly decreased RNA m^6^A levels (Figure 1C), while overexpression of EZH2 in PrEC inversely increased the total m^6^A levels (Figure 1D). Meanwhile, the immunofluorescence (IF) results also indicated much more weakened m^6^A signals in EZH2-deficient PCa cells as compared with control cells (Figure 1E and Supplemental Figure 1A; supplemental material available online with this article; https://doi.org/10.1172/JCI195840DS1), further supporting our conclusion. We next treated PCa cells with a series of EZH2 enzymatic inhibitors GSK126 (25) and EPZ6438 (26) or protein degraders DZNeP (27), MS1943 (28), MS8815 (29), MS8847 (30),and MS177 (17). Notably, either blockade of EZH2’s enzymatic activity or elimination of EZH2 proteins induced a marked m^6^A reduction in both PCa cell lines (Figure 1F and Supplemental Figure 1B), suggesting that the methyltransferase activity of EZH2 alone is enough to modulate m^6^A.

To comprehensively understand the effect of EZH2 on m^6^A regulation, we applied the cutting-edge Nanopore-seq method for genome-wide m^6^A identification. CHEUI, a recently developed algorithm, which enables m^6^A detection at read level, was recruited for data analysis (31). To test the reliability of our model, we first conducted Nanopore-seq in C4-2 cells undergoing METTL3 (the major m^6^A writer) knockdown. As expected, the vast majority of our identified m^6^A sites were hypomethylated upon METTL3 suppression (Supplemental Figure 1C and Supplemental Table 1). In addition, the hypomethylated sites, but not hypermethylated sites, showed a canonical m^6^A enrichment around the stop codon of transcripts (Supplemental Figure 1D), matching the role of METTL3 as a m^6^A writer. Similar to METTL3 suppression, the number of hypomethylated m^6^A sites in EZH2-deficient cells was also significantly higher than the hypermethylated sites (Figure 1G and Supplemental Table 1), which resulted in a global m^6^A downregulation upon EZH2 depletion (Figure 1H). Consistently, only the hypomethylated sites upon EZH2 knockdown could match the conventional m^6^A distribution pattern (Figure 1I), suggesting that EZH2 is more likely to enhance m^6^A methylation. Meanwhile, Gene Set Enrichment Analysis (GSEA) of the gene transcripts with hypomethylated m^6^A sites upon EZH2 deficiency revealed an enrichment towards multiple translation-related pathways (Figure 1J). We next validated the top EZH2-affected m^6^A sites from our Nanopore-seq results by m^6^A CUT&RUN-qPCR assay. As presented in Figure 1K and Supplemental Figure 1E, all tested sites showed dramatic m^6^A decreases in response to EZH2 knockdown in 2 PCa cell lines. Collectively, these data demonstrate that EZH2 functions in upregulating global mRNA m^6^A levels in PCa cells.

### EZH2 activates an m^6^A autoregulation pathway via YTHDF1-METTL14/WTAP signaling.

**Figure 2:**
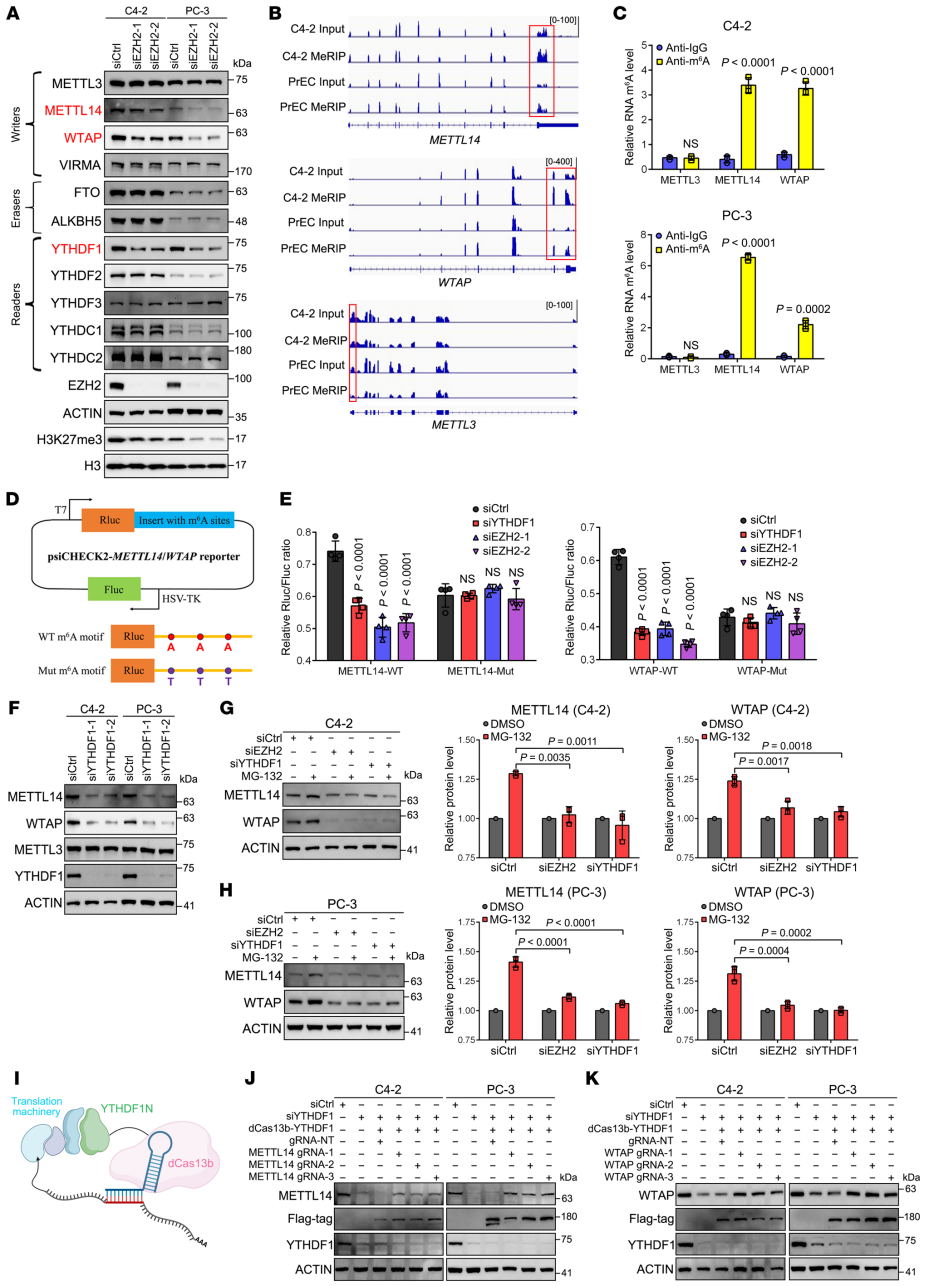
YTHDF1 promotes the translation of METTL14 and WTAP in an m^6^A-dependent manner. (**A**) Western blot to detect the expression change of 11 common m^6^A mediators upon EZH2 suppression in 2 PCa cell lines. (**B**) Genome browser tracks to show the MeRIP-seq data at each indicated loci in C4-2 and PrEC cells, with the peaks around the stop codon and 3′-UTR regions being highlighted. (**C**) The m^6^A CUT&RUN-qPCR assay in 2 PCa cell lines to show the m^6^A enrichment in each indicated transcript. (**D**) Schematic of luciferase reporter constructs. Fragments of METTL14 or WTAP containing 3 predicted m^6^A consensus motifs were cloned downstream of the luciferase coding sequence (labeled as “WT”). In the mutant constructs (“Mut”), the adenosines at these m^6^A consensus sites were substituted with thymidines to disrupt potential m^6^A deposition and YTHDF1 binding. Fluc, firefly luciferase; Rluc, Renilla luciferase. (**E**) Luciferase reporter assay showing relative activity of WT versus mutant constructs in control, EZH2-deficient, or YTHDF1-deficient C4-2 cells. (**F**) Western blot to detect the change of METTL14 and WTAP proteins upon YTHDF1 suppression in 2 PCa cell lines. (**G** and **H**) Control, EZH2-, and YTHDF1-deficient C4-2 (**G**) and PC-3 (**H**) cells were treated with or without Proteasome inhibitor MG-132, followed by Western blot analysis to detect the change of METTL14 and WTAP proteins. Graph showing the relative METTL14 and WTAP protein levels in each indicated group based on 3 biologically independent experiments. (**I**) General overview of the site-specific RNA targeting using dCas13b-YTHDF1N fusion protein, which can trigger the assembly of translation machinery. Created with BioRender.com. (**J** and **K**) YTHDF1-deficient PCa cells were transfected with dCas13b-YTHDF1N and gRNAs targeting METTL14 (**J**) or WTAP (**K**), followed by Western blot analysis to measure the expression change of METTL14 and WTAP, respectively. Since the anti-YTHDF1 antibody we used cannot detect YTHDF1N, the anti-Flag antibody was utilized to capture the dCas13b-YTHDF1N proteins. One-way ANOVA followed by Dunnett’s multiple-comparison test was used for statistical analysis in **E**, **G**, and **H**. Two-tailed Student’s *t* test was used in **C**.

To uncover the mechanism underlying the EZH2-mediated m^6^A regulation in PCa, we first checked the expression change of a set of m^6^A modifiers. As depicted in Figure 2A, the protein level of 3 tested candidates, including m^6^A writers of METTL14 and WTAP as well as m^6^A reader of YTHDF1, were significantly downregulated upon EZH2 suppression. Intriguingly, with respect to transcription level, only the mRNAs of *YTHDF1*, but not *METTL14* and *WTAP*, were coincidentally decreased in response to EZH2 depletion (Supplemental Figure 2, A–C), suggesting that these two m^6^A writers were specifically modulated at the translational level. This finding was reminiscent of our previous report that EZH2 could exert a PRC2-independent function in translational control through fibrillarin (FBL) (14). However, silencing of FBL in PCa cells barely affected the protein levels of METTL14 and WTAP, excluding this possibility (Supplemental Figure 2D). We next shifted the spotlight on the m^6^A intrinsic network. YTHDF1 is known as an m^6^A reader, which promotes the translation initiation of m^6^A-modified mRNAs (32). This information prompted us to examine whether the diminished YTHDF1 expression is directly responsible for the impaired translation of *METTL14* and *WTAP* in EZH2-deficient PCa cells. To this end, we reanalyzed our own methylated RNA immunoprecipitation sequencing (MeRIP-seq) data in both C4-2 and PrEC cells (33). In accordance with our speculation, evident m^6^A peaks could be found around the stop codon of *METTL14* and *WTAP* mRNAs, but were absent in the transcript of *METTL3* (Figure 2B). Moreover, these m^6^A peaks were much higher in C4-2 cells relative to PrEC, correlating with their endogenous EZH2 levels. This observation was further verified by m^6^A CUT&RUN-qPCR assay in C4-2 and PC-3 cell lines (Figure 2C). We next performed RNA immunoprecipitation–qPCR (RIP-qPCR) experiment and confirmed *METTL14* and *WTAP* transcripts as binding targets of YTHDF1 in PCa cells (Supplemental Figure 2E). In addition, treatment of PCa cells with m^6^A inhibitor STM2457 substantially reduced the amounts of YTHDF1-binding *METTL14* and *WTAP* mRNAs (Supplemental Figure 2F), indicating that the YTHDF1-*METTL14*/*WTAP* interaction is m^6^A-dependent. To study the consequence of these YTHDF1-m^6^A recognitions, we used a previously described in vitro luciferase reporter system (Figure 2D) (4). This system contains the m^6^A-enriched fraction of METTL14 or WTAP, with 3 predicted m^6^A sites in each insert. In addition, we mutated the A to T to inactivate the potential YTHDF1 binding. As shown in Figure 2E, depletion of either YTHDF1 or EZH2 efficiently reduced the relative luciferase activity of both WT vectors. However, no significant change of luciferase activity occurred in the mutation reporters (Figure 2E), indicating that the m^6^A modification is critical for the YTHDF1-mediated translation promotion. In line with our above evidence, depletion of YTHDF1 in PCa cells markedly reduced the protein level of both m^6^A writers (Figure 2F), while their mRNA abundance remained unchanged (Supplemental Figure 2G). In comparison, suppression of METTL14 or WTAP in PCa cells had no impact on YTHDF1 expression (Supplemental Figure 2H), further proving our rationale that YTHDF1, but not METTL14 or WTAP, serves as the upstream regulator of this m^6^A intrinsic network.

As the final production of a protein is dictated by both the protein synthesis and degradation rates, we next sought to determine which process is controlled by YTHDF1 to modulate METTL14/WTAP translation. Control, EZH2-, and YTHDF1-deficient PCa cells were treated with either cycloheximide (CHX) to block the de novo protein synthesis, or MG-132 to inhibit proteasomal degradation, followed by immunoblot analyses. In accordance with the role of YTHDF1 in translation promotion, the synthesis of nascent METTL14 and WTAP proteins were significantly suppressed in EZH2- or YTHDF1-deficient cells (Figure 2, G and H), while their protein decay rates were unaltered (Supplemental Figure 2I). To consolidate this finding, we recruited the dCas13b-YTHDF1 system to study the molecular basis behind YTHDF1-mediated translation regulation at *METTL14* and *WTAP* transcripts (34). In brief, the N-terminal domain of YTHDF1 (YTHDF1N) was fused to a catalytically inactive PspCas13b protein, which can target the reader to the RNA of interest by specific guide RNAs (gRNAs) (Figure 2I). Since the C-terminal m^6^A-binding domain of YTHDF1 was fully removed from the fusion protein, this construct allows us to study the m^6^A-initiated downstream effect of YTHDF1 decoupled from its native regulatory context. As revealed in Figure 2, J and K, and Supplemental Figure 2, J and K, when dCas13b-YTHDF1N was recruited to target *METTL14* or *WTAP* mRNAs by gRNAs, the reduced protein level of these 2 m^6^A writers in YTHDF1-deficient PCa cells could be specifically rescued. Together, these results demonstrate that YTHDF1 serves as an upstream regulator of METTL14 and WTAP to control its translation via an m^6^A-dependent pathway.

### Upregulation of FOXA1 by EZH2 enhances YTHDF1 expression transcriptionally.

**Figure 3:**
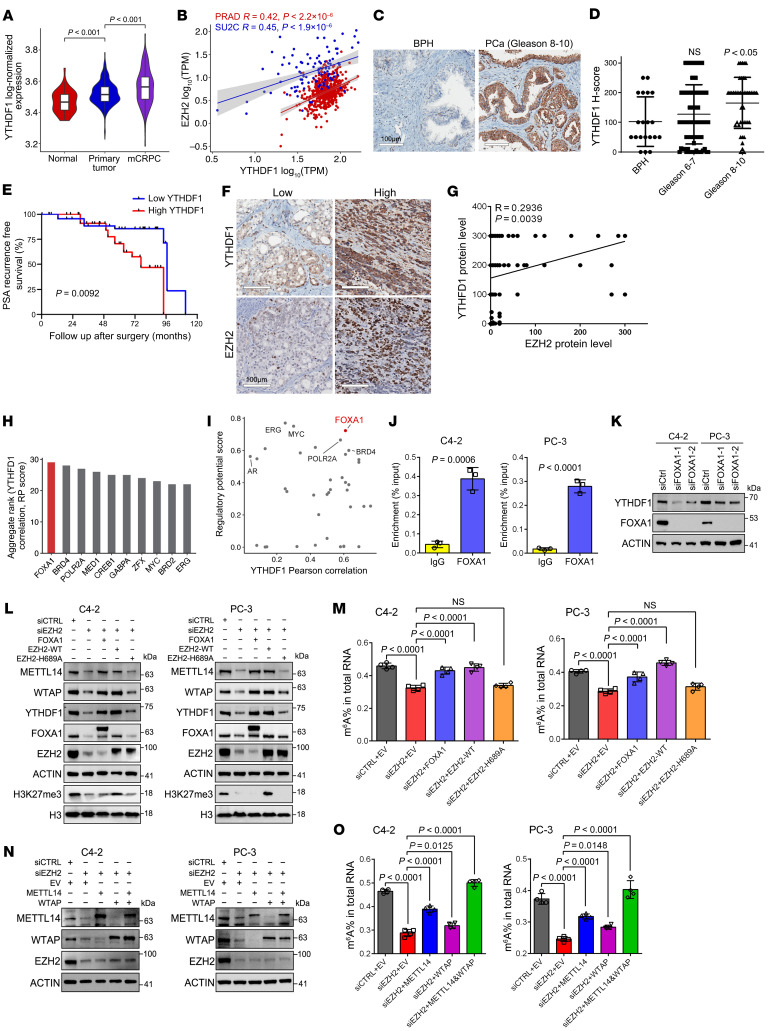
EZH2 activates YTHDF1 transcription through FOXA1 to regulate m^6^A globally. (**A**) Violin plot showing the mRNA level of YTHDF1 in normal (*n* = 52), primary PCa (*n* = 497), and mCRPC (*n* = 101) specimens using data from TCGA and SU2C. *P* values were calculated by 2-tailed Wilcoxon rank-sum test and adjusted for multiple comparisons using Bonferroni correction. mCRPC: metastatic castration-resistant prostate cancer. (**B**) Scatter plot showing the relationship between EZH2 and YTHDF1 mRNA expressions using data from TCGA-PRAD and SU2C, with Spearman correlation coefficient (R) and *P* value as indicated. TPM, transcript per million. (**C**) Representative IHC images of YTHDF1 expression in BPH and high Gleason score PCa tissues as indicated. Scale bar: 100 μm. BPH, benign prostatic hyperplasia. (**D**) Graph showing the YTHDF1 protein levels based on the TMA results from BPH (*n* = 20), Gleason 6–7 PCa (*n* = 83) and Gleason 8–10 PCa (*n* = 44) tissues. (**E**) The association between YTHDF1 expression and PSA recurrence-free survival time of patients with PCa was analyzed by Kaplan-Meier analysis using the patient dataset corresponding to the TMA slide we used. Patients were divided into low (H-score 0–100) and high (H-score ≥ 150) YTHDF1 groups based on the median expression value, which coincided with a natural separation in the frequency distribution. *P* value was calculated by Log-rank (Mantel-Cox) test. PSA, prostate-specific antigen. (**F**) Representative IHC staining of PCa TMA slides using the indicated antibodies. Scale bar: 100 μm. (**G**) Scatter plot showing the correlation between protein levels of EZH2 and YTHDF1, as revealed by the PCa TMA IHC data. The R and *P* value were calculated as indicated. (**H**) Bar chart showing aggregate rank scores for the top 10 transcription factors based on YTHDF1 correlation and Regulatory Potential (RP) Score. (**I**) Scatter plot showing the relationship between YTHDF1 Pearson correlation (*x*-axis) and Regulatory Potential Score (*y*-axis) for various transcription factors, with FOXA1 position highlighted in red. (**J**) ChIP-qPCR assay to monitor the enrichment of FOXA1 at YTHDF1 gene loci in 2 PCa cell lines. (**K**) Western blot to detect the change of YTHDF1 protein level upon FOXA1 knockdown in 2 PCa cell lines. (**L**) Western blot to detect the change of 3 m^6^A mediators upon forced expression of FOXA1, EZH2-WT, or EZH2-H689A in EZH2-deficient PCa cells. (**M**) The m^6^A ELISA was conducted to measure the m^6^A levels in each group of **L**. (**N**) Western blot to validate the reexpression of METTL14 and WTAP in EZH2-deficient PCa cells. (**O**) The m^6^A ELISA was conducted to measure the m^6^A levels in each group of **N**. One-way ANOVA followed by Dunnett’s multiple-comparison test was used for statistical analysis in **D**, **M**, and **O**. Two-tailed Student’s *t* test was used in **J**.

We then aimed to unveil the EZH2-YTHDF1 regulatory network. By searching for both The Cancer Genome Atlas (TCGA) and Stand Up To Cancer (SU2C) databases, we observed that the mRNA level of YTHDF1 was gradually upregulated with the advancement of PCa and positively correlated with that of EZH2 (Figure 3, A and B). IHC assay was next performed using serially sectioned PCa tissue microarray (TMA) slides. Compared with benign prostate controls, PCa specimens with high Gleason scores exhibited significant upregulation of YTHDF1 expression (Figure 3, C and D). Meanwhile, the median survival time of the patient group with high YTHDF1 staining was significantly shorter than that of the group with low YTHDF1 signal (Figure 3E). In agreement with the transcriptomic result, a positive coexpression pattern of YTHDF1 and EZH2 proteins could also be observed (Figure 3, F and G), indicating a strong link between these 2 genes in PCa.

Beyond its canonical role as a transcriptional repressor, EZH2 also functions as a transcription coactivator by interacting with several transcription factors (TFs) (35). Therefore, we speculated that EZH2 may stimulate YTHDF1 transcription via manipulating the latter’s binding TFs. Through interrogating a series of public PCa Chromatin immunoprecipitation–seq (ChIP-seq) profiles, FOXA1 was nominated as the top TF, which is widely distributed throughout the gene body of YTHDF1 (Figure 3, H and I, and Supplemental Figure 3A). Both TCGA and SU2C analyses of PCa specimens revealed a positive correlation between YTHDF1 and FOXA1 mRNA expressions (Supplemental Figure 3B). Meanwhile, the protein levels of both YTHDF1 and FOXA1 are also positively correlated in PCa tissues (Supplemental Figure 3, C and D). Remarkably, emerging evidence has demonstrated that EZH2 could directly methylate FOXA1 protein in PCa cells to protect it from degradation (36, 37), suggesting that FOXA1 might be involved into EZH2-YTHDF1 regulatory axis. In accordance with these reports, silencing of EZH2 by both siRNAs and EZH2 enzymatic inhibitors led to a dramatic downregulation of FOXA1 proteins in PCa cells (Supplemental Figure 3, E and F). Consistently, time-course CHX treatment assay further revealed that either EZH2 knockdown or inhibition of EZH2’s enzymatic activity substantially shortened the half lives of FOXA1 protein (Supplemental Figure 3, G and H). It has been proven that EZH2 monomethylates FOXA1 protein at K295 site to prevent its ubiquitination (36). To recapitulate this finding in our model, PCa cells undergoing either EZH2 knockdown or EZH2 inhibitor treatment were incubated with 10 μM MG-132 for 14 hours and then subjected to coimmunoprecipitation (co-IP) using an anti-FOXA1 antibody. The results showed that EZH2 silencing by both siRNAs and enzymatic inhibitors significantly reduced the FOXA1 K295me1 levels, along with a marked increase of ubiquitinated FOXA1 (Supplemental Figure 3, I and J). To further confirm the relationship between K295me1 and FOXA1 ubiquitination, we overexpressed WT, K295A, or K295R FOXA1 in PCa cells, followed by MG-132 treatment and co-IP assay. As presented in Supplemental Figure 3K, both K295A and K295R mutations markedly boosted the FOXA1 ubiquitination level, indicating an essential role of K295me1 in protecting FOXA1 from degradation. Based on these data, we concluded that EZH2 could stabilize FOXA1 protein in PCa cells through K295me1 deposition.

We then assessed the enrichment of FOXA1 at YTHDF1 gene loci by ChIP-qPCR and confirmed their regulatory relations through Western blot (Figure 3, J and K). To further evaluate the involvement of FOXA1-YTHDF1-METTL14/WTAP signaling axis in the EZH2-mediated m^6^A regulation, we conducted a rescue assay in 2 PCa cell lines. As shown in Figure 3L, overexpression of FOXA1 or EZH2-WT significantly restored the expression of YTHDF1 along with METTL14 and WTAP in EZH2-deficient cells, while forced expression of EZH2-H689A, a catalytically dead mutant, failed to induce the reexpression of all 3 m^6^A modifiers. Consequentially, ectopic expression of FOXA1 or EZH2-WT, but not EZH2-H689A, rescued the decreased m^6^A level in EZH2-deficient PCa cells (Figure 3M). Meanwhile, concurrent reexpression of METTL14 and WTAP in EZH2-deficient cells also achieved a full m^6^A restoration (Figure 3, N and O), further supporting our mechanism. Taken together, our results provide a new model whereby EZH2 controls a previously unappreciated m^6^A autoregulation pathway to enhance the global m^6^A level in PCa cells.

### A PRC2- and m^6^A-dependent function of EZH2 in translational control.

**Figure 4:**
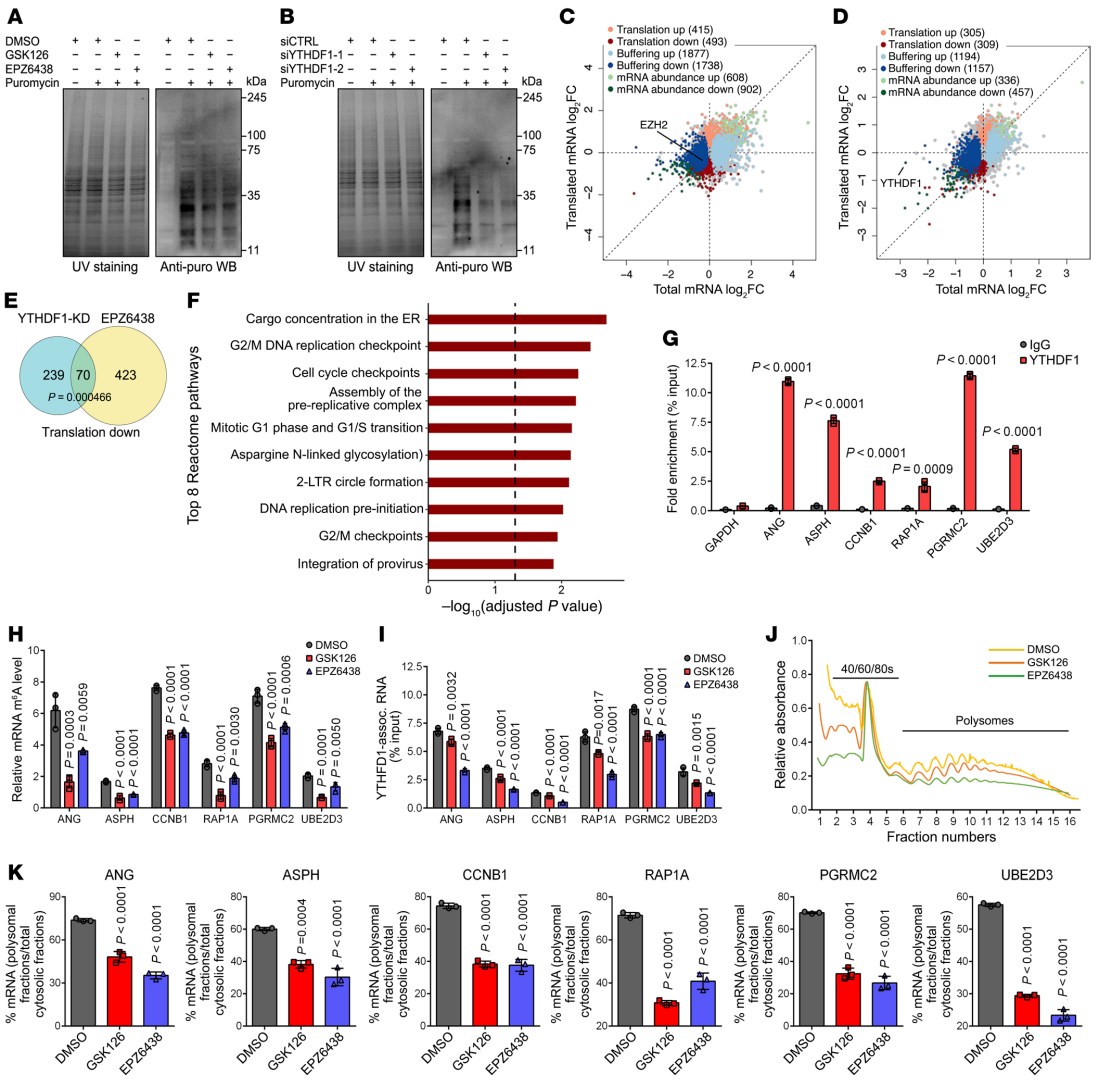
EZH2 exerts a methylation-dependent function in translational control. (**A** and **B**) Puromycylation assay was conducted in C4-2 cells undergoing EZH2 enzymatic inhibitor treatment (5 μM) (**A**) or YTHDF1 deficiency (**B**). Before WB analysis, the whole protein extracts were visualized by UV and shown in the left panel as reference. (**C** and **D**) Scatter plots showing expression changes of mRNA levels and RPFs between control and EPZ6438-treated (**C**) or YTHDF1-deficient (**D**) C4-2 cells. Genes are colored according to their regulation mode. (**E**) Venn diagram showing the overlap between downregulated genes from translation mode after EPZ6438 treatment or YTHDF1 knockdown (KD). (**F**) Gene enrichment analysis of the overlapping genes in **E** using Reactome pathways. Statistical significance was assessed using the hypergeometric test with FDR-corrected *P* values. Only the top 8 enriched pathways are presented. (**G**) RIP-qPCR assay in C4-2 cells to test the binding of YTHDF1 proteins to each mRNA candidate as indicated. (**H**) The m^6^A CUT&RUN-qPCR assay in C4-2 cells upon GSK126 or EPZ6438 treatment to show the m^6^A alterations in each indicated transcript. (**I**) RIP-qPCR assay in C4-2 cells upon GSK126 or EPZ6438 treatment to monitor the change of YTHDF1 binding to each mRNA candidate as indicated. (**J**) Cytoplasmic polysome patterns of DMSO-, GSK126- and EPZ6438-treated C4-2 cells. (**K**) Quantification of the ratio of each polysomal-bound mRNA candidate to the total cytoplasmic mRNA of its own. One-way ANOVA followed by Dunnett’s multiple-comparison test was used for statistical analysis in **H**, **I**, and **K**. Two-tailed Student’s *t* test was used in **G**.

We previously reported a PRC2-independent role of EZH2 in PCa-related translational promotion through activating rRNA 2′-O-methylation and ribosome biosynthesis (14). Intriguingly, our current study indicates that EZH2 may further accelerate the translation efficiency (TE) of a group of m^6^A-modified mRNAs through upregulating YTHDF1 expression in a PRC2- and lysine trimethylation (K_me3_)-dependent manner. To test this notion, we first performed the puromycylation assay to monitor the global protein synthesis by labelling the nascent peptides with puromycin (38). Treatment of C4-2 cells with EZH2 enzymatic inhibitors of GSK126 and EPZ6438 both induced a broad decrease of de novo protein synthesis without affecting EZH2 expressions (Figure 4A and Supplemental Figure 4A), proving that the methyltransferase activity of EZH2 has an impact on translation. Similarly, depletion of YTHDF1 also led to a reduced production of newly synthesized proteins (Figure 4B and Supplemental Figure 4B), matching its established role in enhancing translation (32).

To systematically understand the K_me3_ & m^6^A-dependent role of EZH2 in TE regulation, we utilized RiboLace to capture the ribosome-protected fragments (RPFs) in C4-2 cells undergoing EPZ6438 treatment or YTHDF1 deficiency for sequencing. The mRNA abundance in each group was measured in parallel by RNA-seq. As an optimized approach of the traditional Ribosome sequencing (Ribo-seq), RiboLace requires much less starting material and avoids the tedious ultracentrifugation steps (39). By conjoint analyses of RiboLace and RNA-seq data, differential genes were divided into 3 groups of (a) “translation”, genes with evident change in RPF but not in mRNA; (b) “buffering”, genes with changes in mRNA without corresponding changes in RPF; (c) “mRNA abundance”, genes with changes in RPF that keep pace with that in mRNA.

Upon EPZ6438 treatment, 908 genes were identified in the translation group; 3,615 in the buffering group; and 1,510 in the mRNA abundance group (Figure 4C and Supplemental Table 2). Meanwhile, depletion of YTHDF1 led to 659 genes in the translation group; 2,351 in the buffering group; and 793 in the mRNA abundance group (Figure 4D and Supplemental Table 2). The expression changes in each group matched the desired setting, supporting the effectiveness of the classification algorithm (Supplemental Figure 4C). By comparing the EPZ6438 treatment RiboLace data with our previously obtained Ribo-seq results in EZH2-knockdown C4-2 cells, we observed a much lower gene number in the translation group (908 versus 2,687), while the size of mRNA abundance group remained in the same scale (1,510 versus 1,548) (14). These data indicated that both the PRC2-dependent and -independent roles of EZH2 contribute to the translational control, while its enzymatic activity is solely responsible for the transcriptional regulation. To evaluate the coregulatory function of EZH2 and YTHDF1 in TE, we recruited the translation-down and mRNA abundance-down subsets from both conditions for analysis. According to the RiboLace classification, the translation-down subgroup contains genes with impaired TE, while genes in the mRNA abundance-down subgroup are solely repressed at the transcriptional level. In line with our prediction, genes in the translation-down subgroups of EPZ6438-treated and YTHDF1-deficient cells were significantly overlapped and coenriched in multiple cellular pathways (Figure 4, E and F, *P* < 0.001, hypergeometric test). Meanwhile, the overlap between the 2 mRNA abundance-down subgroups was not statistically significant. (Supplemental Figure 4D; *P* = 0.24, hypergeometric test). These data demonstrate that EZH2 and YTHDF1 tend to share a function in governing the translation, but not the transcription process.

To validate the RiboLace results, we focused on the 70 overlapping genes that showed diminished TE in response to both EPZ6438 treatment and YTHDF1 deficiency in C4-2 cells (Figure 4E). These genes are deemed to be regulated by EZH2 solely at the translation level via targeting of YTHDF1 in a m^6^A-dependent manner. As expected, 45 out of the 70 genes contain m^6^A sites within their transcripts, as revealed by our Nanopore-seq and MeRIP-seq data (Supplemental Table 3). Most of their m^6^A sites are located within the 3′-UTR region and conform to the consensus DRACH motif (Supplemental Figure 4, E and F). We then selected 6 candidates for in-depth investigation, since they are all cancer-related genes with at least one m^6^A site in mRNA. The binding of YTHDF1 to these candidates was first confirmed by RIP-qPCR assay (Figure 4G). Upon GSK126 or EPZ6438 treatment, the m^6^A levels in all candidates were significantly decreased, along with their YTHDF1 binding intensity (Figure 4, H and I). These data suggested that all candidates are subjected to the EZH2-YTHDF1-m^6^A regulation in PCa cells. To further unveil the impact of EZH2’s enzymatic activity on the TE of these genes, we performed polysome profiling analysis and found that GSK126 or EPZ6438-treated C4-2 cells exhibited a much lower profiling than the control sample (Figure 4J), which is largely identical to the pattern upon YTHDF1 silencing (Supplemental Figure 4G). We then collected the polysome fractions to measure the mRNA distribution of each candidate. The results showed that both GSK126/EPZ6438 treatment and YTHDF1 knockdown significantly impaired the amount of each candidate’s mRNA that bound to polysomes (Figure 4K and Supplemental Figure 4H). In summary, the above results provide evidence that the enzymatic activity of EZH2 is capable of profoundly reshaping the TE pattern in PCa cells through targeting of YTHDF1.

### YTHDF1 as a key determinant for EZH2 to exert its oncogenic functions.

**Figure 5:**
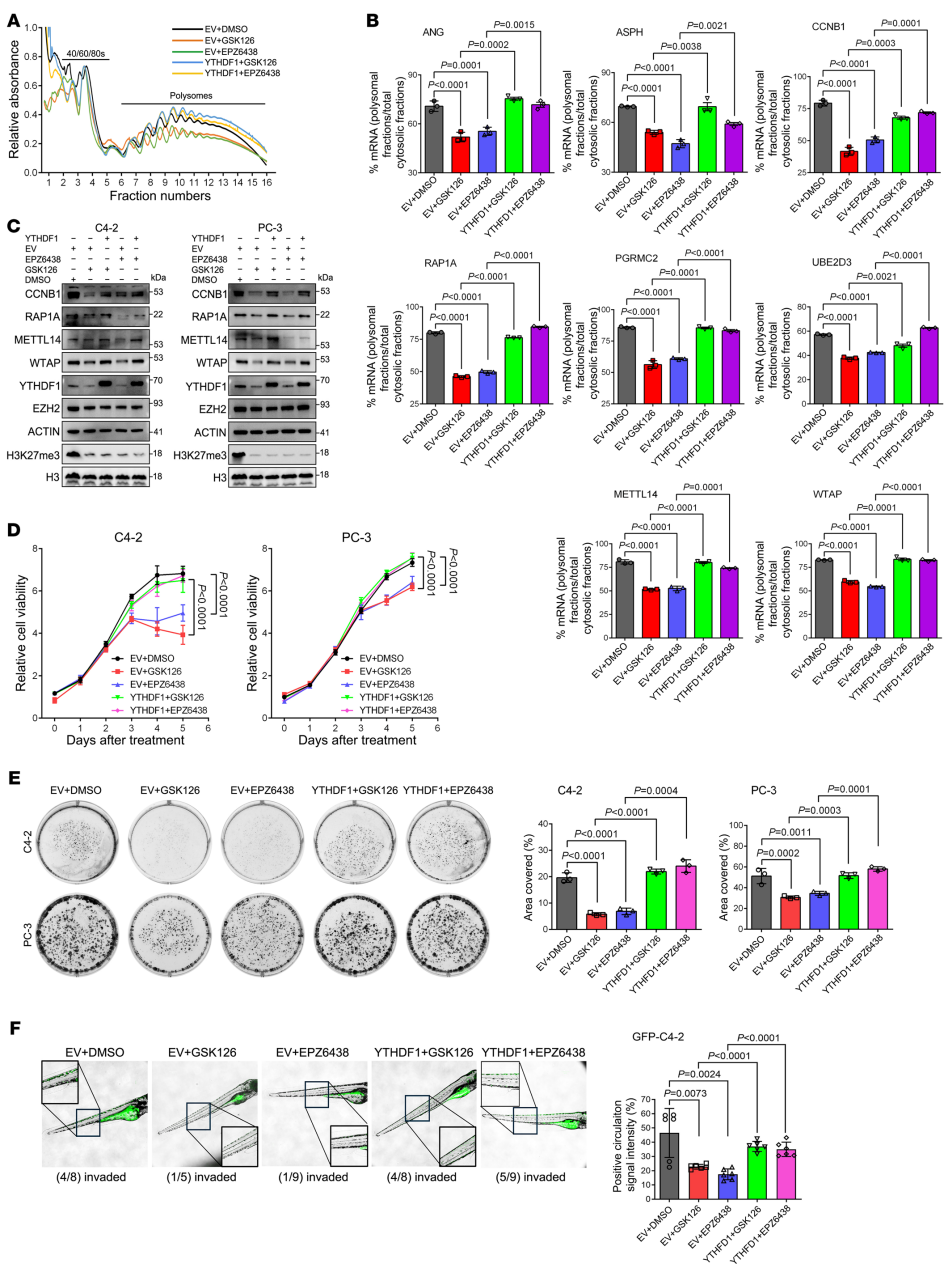
EZH2 enzymatic inhibitors suppress PCa progression through YTHDF1. (**A**) Cytoplasmic polysome pattern of C4-2 cells in each group as indicated. (**B**) Quantification of the ratio of indicated polysomal-bound mRNAs to the total cytoplasmic mRNA of their own. (**C**) Western blot to detect the expression of CCNB1, RAP1A, METTL14, and WTAP in each group of 2 PCa cell lines as indicated. (**D**) Cell viability assay to assess the proliferative capacity of GSK126- or EPZ6438-treated PCa cells (5 μM for each) overexpressing YTHDF1. (**E**) Colony formation assay was performed in each group as indicated. Graphs showing the percentage of the area in each well covered by crystal violet–stained cell colonies. (**F**) GFP-labeled C4-2 cells in each condition were injected into zebrafish embryos. Tumor cell invasion was examined upon 3 days and images were taken under 4× magnification. Embryos exhibiting positive circulation signals were classified as “invaded.” Graph showing the mean fluorescence intensity (%), with individual data points representing each measurement. For each group, 3 zebrafish larvae were randomly selected, and 2 distinct regions per larvae were analyzed to measure fluorescence intensity. One-way ANOVA followed by Dunnett’s multiple-comparisons test was used in panels **B**, **E**, and **F** when multiple groups were compared. For comparisons involving only 2 groups, an unpaired 2-tailed Student’s *t* test was applied (**B**, **E**, and **F**). In **D**, statistical significance was assessed using an unpaired 2-tailed Student’s *t* test at the final timepoint.

A number of studies have proven that EZH2 canonically drives cancer progression through epigenetic silencing of tumor suppressive genes via catalyzing H3K27me3 (40). Remarkably, our above evidence supports a notion that EZH2 may further facilitate oncogenesis via enhancing the translation of a series of cancer regulators through its catalytic activity, with YTHDF1 playing a pivotal role in this process. To assess this, we first confirmed that suppression of YTHDF1 substantially reduced the proliferative rate of PCa cells (Supplemental Figure 5A), along with their migratory and invasive capabilities (Supplemental Figure 5, B and C). Interestingly, when comparing the polysomal profiles, we noted that forced expression of YTHDF1 in C4-2 cells could significantly overcome the decreased proportion of polysomes induced by GSK126 or EPZ6438 (Figure 5A). In addition, YTHDF1 overexpression restored the mRNA amount of all the above examined TE-affected genes on polysomes (Figure 5B). METTL14 and WTAP, 2 key downstream targets of YTHDF1 in our model, also exhibited an evidently reduced mRNA enrichment in polysomal fractions upon GSK126 or EPZ6438 treatment, and the impairment was robustly rescued upon YTHDF1 reexpression (Figure 5B). Among all of these tested candidates, CCNB1, RAP1A, METTL14, and WTAP are previously validated prostatic oncogenes (6, 41–43). As expected, all 4 oncogenes showed an alteration in protein level that matched their TE changes, supporting the essential role of YTHDF1 in EZH2-mediated oncogenesis (Figure 5C). Consistently, reexpression of YTHDF1 evidently reversed the repressed proliferation in PCa cells incubated with GSK126 or EPZ6438 (Figure 5D). Colony formation assays also showed that the abolished PCa cell growth upon long-term treatment of GSK126 or EPZ6438 was restored by concurrent expression of ectopic YTHDF1 (Figure 5E). To recapitulate these findings in vivo, we conducted a zebrafish embryo metastasis assay using GFP-labeled PCa cells and observed the same tendency (Figure 5F and Supplemental Figure 5D). Above all, our data support YTHDF1 as a critical downstream effector of EZH2 to promote prostate carcinogenesis.

### Targeting EZH2 and m^6^A synergistically kills PCa tumors.

**Figure 6:**
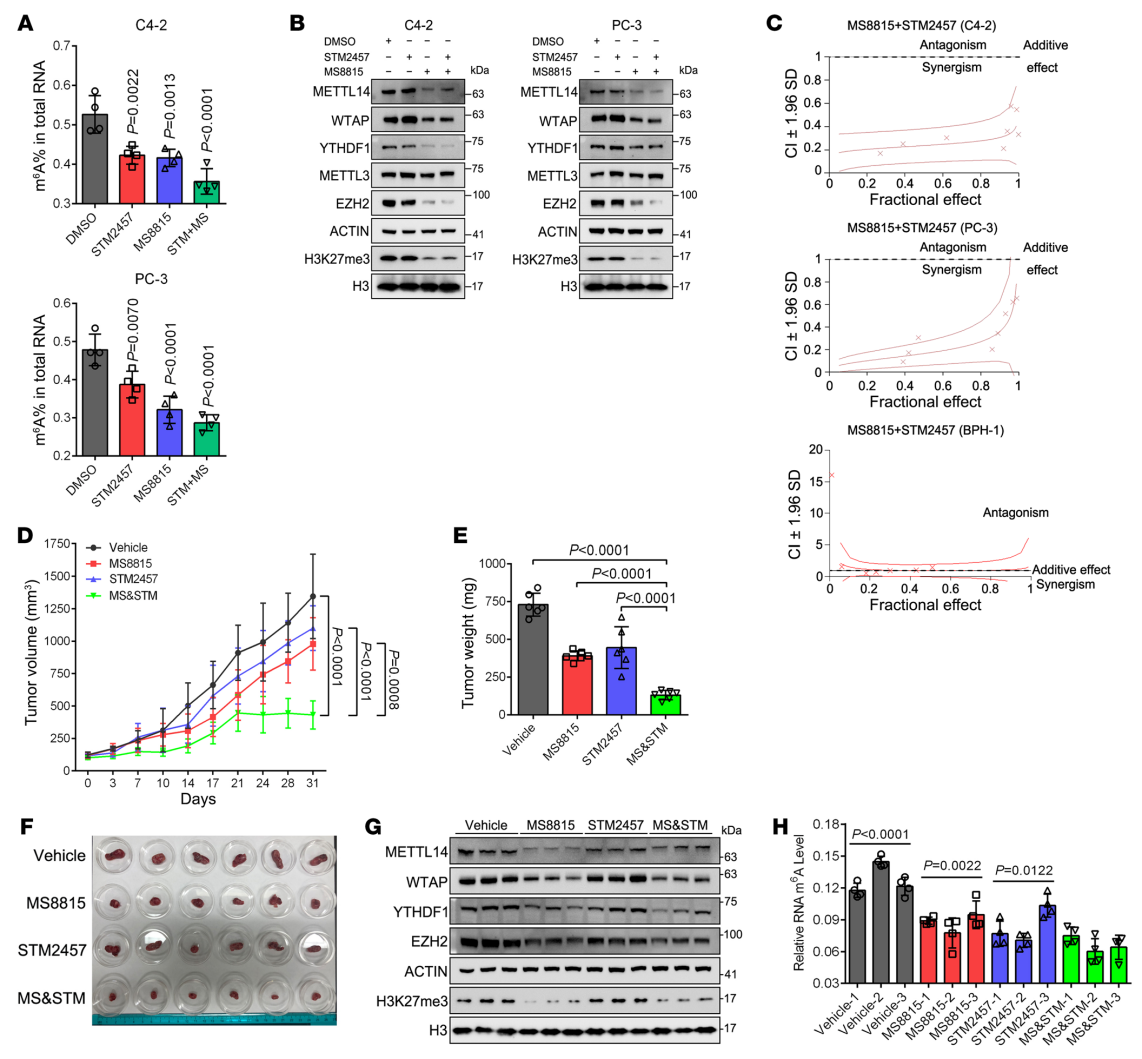
Combinational targeting of EZH2 and m^6^A achieves a synergistic effect in treating aggressive PCa. (**A**) The m^6^A ELISA to measure the global m^6^A level in 2 PCa cell lines treated with STM2457 (5 μM), MS8815 (1 μM), or combination. (**B**) Western blot to detect the expression of indicated proteins in each group of **A**. (**C**) Representation of combination index plot of STM2457 combined with MS8815 in 2 PCa cell lines and BPH-1 cells. Doses below the dotted line represent the synergistic effect, while doses above the dotted line indicate the antagonistic effect. (**D**–**F**) The LuCaP 35CR PDX tumors were implanted subcutaneously into NCG mice, followed by delivery of STM2457, MS8815, or combination. Tumor volume was measured by caliper twice a week and plotted in **D**. Day 0 corresponds to the time point when the tumor volume reached approximately 100 mm^3^, at which time drug administration was initiated. At the end point of measurement, tumors were harvested, weighed (**E**), and pictured (**F**). Data represent Mean ± SD from *n* = 6 mice in each group. (**G** and **H**) Three randomly selected tumors from **F** were subjected to WB analysis to detect the expression of three m^6^A mediators as indicated (**G**), along with m^6^A ELISA to measure the m^6^A changes (**H**). One-way ANOVA followed by Dunnett’s multiple-comparison test was used for statistical analysis in **A**, **D**, **E**, and **H**.

Targeting of m^6^A and its regulators has emerged as a promising avenue for anticancer therapy (44). As the first-in-class small-molecule m^6^A inhibitor, STM2457 selectively reduces RNA m^6^A levels by disrupting the METTL3-METTL14 interaction (45). As expected, STM2457 reduced the total m^6^A level in PCa cells without affecting the expression of two m^6^A enzymes (Supplemental Figure 6, A and B). Although lower than that in BPH-1 cells, the half-maximal inhibitory concentration (IC_50_) of STM2457 was still above 15 μM in 2 PCa cell lines, indicating a modest inhibitory effect (Supplemental Figure 6C). We then expected that EZH2 inhibitors may strengthen the efficacy of STM2457 in treating EZH2^high^ m^6^A^high^ tumors (i.e., advanced PCa types) by further elimination of m^6^A via downregulation of YTHDF1/METTL14/WTAP along with blocking the multifaceted tumorigenic functions of EZH2. MS8815, a recently discovered EZH2 proteolysis targeting chimera (PROTAC) degrader, was employed for EZH2 elimination in our model (29). Remarkably, dual treatment of STM2457 and MS8815 in PCa cells achieved a more dramatic m^6^A reduction as compared with single drug use (Figure 6, A and B). Furthermore, combinatorial use of STM2457 and MS8815 reached an evident synergistic effect in killing PCa cells (Figure 6C). In comparison, these 2 drugs only created an additive effect in BPH-1 cells (Figure 6C), suggesting that the synergy tends to be limited in EZH2^high^ m^6^A^high^ models. To further test this strategy in vivo, we delivered STM2457 and MS8815 into male mice bearing LuCaP 35CR, an advanced castration-resistant PCa (CRPC) patient-derived xenograft (PDX) (46). In accordance with our in vitro observations, combinational treatment of STM2457 and MS8815 reduced the tumor burden more effectively when compared with those utilizing one drug only (Figure 6, D–F). The follow-up analyses of the resulting tumors confirmed the on-target drug effects in reducing EZH2, YTHDF1, and m^6^A levels (Figure 6, G and H, and Supplemental Figure 6D). In conclusion, these data demonstrated that combinational targeting of EZH2 and m^6^A activities may serve as a more effective approach over EZH2 targeting only for advanced PCa therapy.

## Discussion

Numerous histone modifiers have been proved to be crucial determinants of m^6^A methylation, which control its precise and dynamic deposition in the transcriptome (18). Here, we focus on EZH2, a critical histone methyltransferase, and disclose its previously unknown role in m^6^A regulation. We document that EZH2 sustains a hyper-m^6^A state in PCa cells through its lysine methylation activity to promote oncogenesis and survival. Notably, in this study, we employed Nanopore-seq technology to directly map EZH2-mediated m^6^A modifications at the individual transcript level. In contrast with the conventional m^6^A detection techniques, Nanopore-seq enables the measurement of m^6^A stoichiometry at single-base resolution and avoids the biases introduced during PCR steps (1). While Nanopore-seq offers superior resolution, antibody-based experiments such as MeRIP-seq can complement m^6^A detection with broader coverage of m^6^A sites across the transcriptome (47). Thus, to leverage the strengths of both techniques, we integrate results from Nanopore-seq with our previous MeRIP-seq dataset for our analysis (33).

The mechanism underlying the EZH2-mediated m^6^A regulation is complex. Instead of targeting one single m^6^A intrinsic factor or modulating one unique m^6^A-related process, EZH2 elevates the global m^6^A level in PCa cells through triggering an YTHDF1-mediated m^6^A autoregulatory pathway. It is noteworthy that our study is not the sole example showing the existence of an autoregulatory loop in m^6^A machinery. A recent paper confirmed that many of the core components in m^6^A signaling were extensively methylated by m^6^A in mouse embryonic stem cells (mESCs) (48). Among them, the transcript of m^6^A reader Ythdc1, an arbiter of splicing events, underwent dramatic splicing and expression change in response to acute depletion of Mettl3 in an m^6^A-dependent manner. These data underscore the importance of m^6^A self regulation in the control of alternative splicing. In agreement, we observe evident m^6^A peaks in the transcripts of *METTL14* and *WTAP*, which could be recognized by YTHDF1 to augment translation. Conceivably, this signaling is frequently hijacked by tumor cells to create a favorable environment during oncogenesis.

In the present study, YTHDF1 was characterized as a key m^6^A reader, which is targeted by EZH2 to promote PCa tumorigenesis. Compelling evidence has shown that multiple oncogenes relevant to PCa can be activated by YTHDF1 in an m^6^A-dependent manner, thereby enhancing tumor progression (49–51). Building upon these findings, we further demonstrated that YTHDF1 could profoundly reshape the PCa m^6^A landscape by initiating an m^6^A autoregulatory pathway. Notably, a recent publication also highlighted YTHDF1 as a central m^6^A reader in PCa (52). By analyzing the epitranscriptomes of 162 localized prostate tumors, YTHDF1 was identified as the most prominently upregulated YTHDF family member in PCa, with a clear association with adverse clinical features and a key role in promoting the translation of oncogenic mRNAs. In contrast with YTHDF1’s predominant role in enhancing translation, its paralogs YTHDF2 and YTHDF3 primarily function to promote the decay of m^6^A-modified transcripts (53). Although the protein levels of YTHDF2 and YTHDF3 remains unaltered following EZH2 depletion (Figure 2A), it is possible that functional redundancy or compensatory mechanisms exist among these family members. Thus, it would be valuable to explore whether EZH2 could influence the fate of m^6^A-marked transcripts through coordinated regulation of all 3 YTHDF proteins in a PCa model.

Our findings further suggest that EZH2 methylates FOXA1, but not histone H3, to govern YTHDF1 expression. As a pioneer TF, FOXA1 is usually considered to play essential roles in androgen-dependent PCa, since it induces open chromatin conformations to facilitate androgen receptor (AR) binding (54). Intriguingly, our current study has confirmed the FOXA1-mediated YTHDF1 upregulation in both AR-dependent C4-2 and AR-independent PC-3 cell lines. This observation is not surprising, since FOXA1 is still able to boost the binding of other TFs to the opened YTHDF1 nucleosomal region in the AR-negative PCa models (55).

Beyond our previous report regarding the PRC2-independent function of EZH2 on translational regulation (14), this study demonstrates that EZH2 could also accelerate the translation process in PCa cells through its enzymatic activity. Coincidently, both of EZH2’s functions are achieved by intertwining with RNA modifications. In our former model, EZH2 upregulates the rRNA 2′-O-methylation status through interaction with FBL and thus imposes a global impact on the translation process. In contrast, the influence of EZH2-m^6^A crosstalk on TE regulation is largely restricted in m^6^A-modified transcripts with YTHDF1-binding capacities. A schematic illustration was presented in Supplemental Figure 6E to demarcate the dual roles of EZH2.

Despite the success of small-molecule m^6^A inhibitor STM2457 in treating blood cancers, its effectiveness against solid PCa tumors remains poor (56, 57). In parallel, the EZH2-targeting strategy also showed limited efficacy in curing patients with aggressive PCa (58). Here, we demonstrate that the combination administration of STM2457 with EZH2 degrader MS8815 elicits much more potent effects in suppressing PCa. Considering the wide impact of both EZH2 and m^6^A on transcriptional and posttranscriptional regulations, the detailed mechanism of action behind this synergistic effect along with its downstream network await further investigation. Overall, the current work expands our knowledge regarding the convergence of a key histone modifier and a widespread RNA modification type, which may inspire a therapeutic advance in treating EZH2-dependent cancers.

## Methods

### Sex as a biological variable.

Our study exclusively examined male mice because the disease modeled is only relevant in males.

### Cell lines and PDX model.

Human PCa cell line C4-2 was provided as a gift from Leland Chung (Cedars-Sinai, Los Angeles, California, USA), while PC-3, 22RV1, C4-2B, DU145, LNCaP, abl, and VCaP were purchased from ATCC. Human benign prostatic hyperplasia cell line BPH-1 was a kind gift from Xuesen Dong (University of British Columbia). All prostate cell lines were grown in RPMI 1640 medium supplemented with 10% FBS and authenticated periodically using short tandem repeat (STR) profiling. Human Prostate Epithelial Cells (PrEC) were purchased from Lonza and cultured using PrEGM prostate epithelial cell growth medium bullet kit (Lonza). All cell lines and primary cells were cultured at 37°C in a humidified atmosphere of 5% CO_2_. All cells were routinely tested for Mycoplasma contamination. The LuCaP 35CR patient-derived xenograft (PDX) was kindly provided by Eva Corey (University of Washington, Seattle, Washington, USA).

### Transfection of siRNAs and plasmids.

All silencer-select siRNAs used in this study were purchased from Thermo Fisher (siEZH2-1: s4916, siEZH2-2: s4917; siYTHDF1-1: s29743, siYTHDF1-2: s29744; siFOXA1-1: s6687, siFOXA1-2: s6688; siFBL-1: s4820, siFBL-2: s4821; siMETTL3-1: s32141, siMETTL3-2: s32142, siMETTL14: s33679, siWTAP: s18431). In addition, one siRNA targeting the 3′-UTR of endogenous EZH2 (sequence: 5′-UUGCCUUCUCACCAGCUGC-3′) and one siRNA targeting the 3′-UTR of endogenous YTHDF1 (sequence: 5′-ATCGGTCTAAAGTGCTAATTT-3′) were synthesized by Thermo Fisher for the rescue assays.

For siRNA transfection, Lipofectamine RNAiMAX (Invitrogen) were utilized according to the procedure of manufacturer. Meanwhile, plasmid transfection was achieved using Lipofectamine 3000 (Invitrogen) by following its protocol. Medium was replaced after 24 hours and cell samples were collected at 2–3 days posttransfection.

### Treatment of cells with inhibitors.

EZH2 inhibitors of MS1943, MS8815, MS8847, and MS177 were discovered and kindly provided by Jian Jin (Mount Sinai, New York, New York, USA); GSK126 was purchased from BioVision; DZNep and EPZ6438 were purchased from Selleck Chemicals. Meanwhile, m^6^A inhibitor STM2457 was ordered from MedChemExpress. PCa cells were treated with inhibitors at the indicated concentrations for 3 days until further use.

### Measurement of m^6^A by ELISA.

The EpiQuik m^6^A RNA Methylation Quantification Kit (EpiGentek) was used to quantify the m^6^A ratio in total RNA. For each sample, 200 ng RNA was mixed with binding solution and bound to strip wells. The m^6^A signals were then captured using a specific antibody. The detected signals were further enhanced and quantified colorimetrically by reading the absorbance in a Tecan plate reader at a wavelength of 450 nm. The amount of m^6^A was proportional to the OD intensity measured.

### dPspCas13b assays.

The cmv-d0-dPspCas13b-GGS-NYTHDF1 plasmid was a gift from Bryan Dickinson (University of Chicago, Chicago, Illinois, USA) (Addgene plasmid # 119855), while the PspCas13b gRNA backbone vector was a gift from Feng Zhang (Broad Institute, Cambridge, Massachusetts, USA) (Addgene plasmid # 103854). The gRNAs targeting indicated genes or control gRNA were cotransfected with dPspCas13b-YTHDF1N into control or YTHDF1-deficient PCa cells. Cells were harvested at 2 days posttransfection and subjected to WB and RT-qPCR analyses. All gRNA oligo sequences could be retrieved in Supplemental Table 5.

### Dual-luciferase assay.

The psiCHECK-2 plasmid was purchased from Promega. The reporter vectors were generated by subcloning the 500 bp m^6^A-containing sequence of METTL14 or WTAP (both WT and A-to-T mutant) into the psiCHECK-2 vector. For METTL14, 3 m^6^A sites of *chr4:118,710,312*, *chr4:118,710,376*, and *chr4:118,710,561* were included. For WTAP, 3 m^6^A sites of *chr6:159,755,381*, *chr6:159,755,543*, and *chr6:159,755,577* were included. Dual-luciferase assays were performed in C4-2 cells at 24 hours posttransfection using Dual-Glo luciferase reagent (Promega) according to the manufacturer’s instructions and a Tecan plate reader.

### m^6^A CUT&RUN assay.

To detect the m^6^A change in a specific mRNA region, the m^6^A CUT&RUN assay was conducted by using the EpiNext CUT&RUN RNA m^6^A-Seq Kit (EpiGentek). For each sample, 10 μg of total RNA was submitted for m^6^A RNA enrichment by following the user’s guide. The obtained RNA fragments were reverse transcribed into cDNA using Maxima H Minus First Strand cDNA Synthesis Kit (Thermo Fisher), and the relative m^6^A level was then calculated by RT-qPCR analysis. All CUT&RUN primers used here were summarized in Supplemental Table 5.

### Nanopore-seq and data analysis.

The Nanopore-seq library was prepared as described previously (59). The total RNA was first extracted using RNeasy Plus Mini Kit (Qiagen), followed by mRNA purification using PolyATtract mRNA Isolation System (Promega). The mRNA concentration was measured by Qubit RNAHS assay kit (Thermo) and the quality was monitored by Agilent Bioanalyzer RNA Pico assay chip. Then, 500 ng mRNA was subjected to library construction using an Oxford Nanopore direct RNA sequencing kit (SQK-RNA002) according to the manufacturer’s manual. The resulting library was sequenced on an R9.4.1 flow cell (FLO-MIN106D) using a MinION sequencer, and the FAST5 raw sequencing data was obtained in a real-time manner.

For data analysis, Nanopore electrical signals were converted to FASTQ using the guppy base caller (v6.0.1) and reads were aligned to the human transcriptome using minimap2 and direct RNA-seq parameters (“-ax splice -uf -k14”, v2.24) with the GENCODE v42 annotation. Signal data was matched with alignment data using nanopolish (v0.14.0). CHEUI was then used to call m^6^A modification status for DRACH motifs using default parameters (31). A position within a transcript was called methylated if at least 50% of reads were methylated at this position.

### Puromycylation assay.

The puromycylation assay was conducted as described before (38). PCa cells were pretreated with CHX (Sigma) at a concentration of 355 μM for 15 minutes to freeze polysomes. Then, puromycin (Sigma) was added into the culture medium to a final concentration of 91 μM. After treatment for 5 minutes at 37°C, the protein samples were immediately prepared and puromycin incorporation into the nascent chain was detected by WB using a specific antipuromycin antibody (Supplemental Table 4). The Stain-Free Precast Gels (Bio-Rad) were used for the UV-induced visualization of total proteins in each sample.

### RiboLace and RNA-seq.

C4-2 cells in both drug-treated and siRNA-knockdown groups were incubated with 100 mg/mL CHX (Sigma) for 5 minutes. Then, cells were spun down and washed with ice-cold PBS. Cell pellets were flash-frozen with liquid nitrogen and stored at –80°C until use. The RiboLace and RNA-seq library constructions were completed using ALL-IN-ONE RiboLace Gel Free Kit (IMMAGINA) by following its protocol. The obtained libraries were then subjected to Admera Health for sequencing.

The RNA-seq read processing and quality control were performed using the nf-core/rnaseq pipeline (v3.15.0) (60). Briefly, reads were aligned to the human reference genome hg38 using STAR (v2.7.10a) (61) and raw read counts were obtained using featureCounts (v2.0.3) (62) using GENCODE v42 annotation. Anota2seq (v1.26) (63) was used to normalize raw read counts in parallel with RiboLace data using TMM-log_2_ method.

Raw reads were first trimmed using Cutadapt (v4.2) to remove LACE-seq 3’ linker sequences, retaining sequences longer than the minimum ribosome protected fragment length (20 nts) plus UMI sequence length. UMI sequences were then extracted using umi_tools (v1.1.5) (64). Sequences of rRNA, tRNA, and other ncRNA were obtained from RNAcentral (65), and RiboLace reads were aligned to these ncRNAs using Bowtie2 (v2.5.4) (66). Unaligned reads were then mapped to the human reference genome hg38 using STAR (v2.7.10a) and deduplicated based on UMI sequences using umi_tools. Raw read counts were obtained using featureCounts (v2.0.3) using GENCODE v42 annotation.

RNA-seq and RiboLace count data were integrated using anota2seq (v1.26). Count data was normalized using TMM-log_2_ method and normalized RPF data was batch corrected using ComBat from the sva R package (v3.52) between YTHDF1 and EPZ6438 treatment experiments. Anota2seq categorized each gene as background, mRNA abundance, translation, or mRNA buffering groups with *P* < 0.01 and FDR < 0.15 cutoffs. GO analysis was achieved using g:Profiler web server (67) with gene sets less than 2,000 genes in size. Overlap analysis was performed using phyper R function, and overlap genes were compared with all genes that showed detectable changes by Anota2seq.

### Polysome profiling.

C4-2 cells in each group were incubated with 100 μg/mL CHX (Sigma) for 15 minutes at 37°C and then lysed in polysome extraction buffer (20 mM Tris-HCl, pH 7.5, 100 mM KCl, 5 mM MgCl_2_, 0.3% IGEPAL CA-630). Nuclei and debris were pelleted by centrifugation at 10,000 rpm for 10 minutes at 4°C. The supernatant containing ribosomal particles was then separated on a 10%–50% sucrose gradient by ultracentrifugation at 200,000*g* for 1.5 hours at 4°C. The ribosome distribution was analyzed using BioComp fractionation and analysis system. RNA from each fraction was isolated with TRIzol (Invitrogen) and subjected to RT-qPCR assay.

### Zebrafish embryo metastasis assay.

Zebrafish were maintained at Northwestern University in a fully automated recirculating system under standard laboratory conditions. Adult fish were housed in 4 L or 8 L tanks at a density of no more than 7 fish per liter. The water was maintained at 26–28°C with a pH of 7.0–7.5 and a 14-hour light/10-hour dark cycle. Water quality was ensured through continuous mechanical and biological filtration with multiple daily water exchanges and weekly monitoring. Fish were fed a mixed diet 2 to 3 times daily, and their health status was monitored visually each day.

Approximately 100 GFP-labeled PCa cells were microinjected into the perivitelline space of 48-hours postfertilization (hpf) embryos using a Tritech Research PL1-100 micromanipulator and a pneumatic injector. Embryos that were incorrectly injected into the yolk sac were excluded from the subsequent analysis. After injection, embryos were washed, transferred to 6-well plates, and incubated at 34°C in E3 medium. On day 3 postinjection (5 days postfertilization), larvae were examined under a fluorescence microscope to assess cancer cell dissemination. Larvae displaying GFP-positive cells outside the injection site, particularly within the vasculature or tail region, were classified as having metastatic invasion. Fluorescent signal intensity of circulating tumor cells was quantified using ImageJ.

### Xenograft study.

One week after castration, mice were implanted subcutaneously with LuCaP 35CR tumor bits. To minimize baseline variability, mice were divided into treatment groups based on body weight when tumor volume reached 100 mm^3^. The sample size for each group (*n* = 6) was chosen based on experimental feasibility and ethical considerations. Mice were given vehicle, MS8815 (50 mg/kg, 100 μL × twice by intraperitoneal injection), STM2457 (50 mg/kg, 100 μL × twice by oral gavage), and a combination of MS8815 and STM2457. Blinding was not used in the animal studies. The tumor volume was measured using calipers using the formula (L×W×W/2). Tumor volume and body weight were measured twice weekly. Animals were treated by oral gavage or intraperitoneal injection on a weekly schedule of 5 days on, 2 days off. After 30 days of treatment, mice were euthanized and tumors were excised and weighed.

### Statistics.

Statistical analysis was performed using GraphPad Prism (version 6.0) or R (version 4.4.3) and presented as mean ± SD. For comparisons between 2 groups, 2-tailed Student’s *t* test was used. For experiments with more than 2 groups, 1-way ANOVA was used followed by Dunnett’s test. Gene enrichment analysis was performed using hypergeometric tests. Statistical data were considered significant if *P* < 0.05. The results were reproducible and conducted with established internal controls. When feasible, experiments were repeated 3 or more times and yielded similar results. We have indicated the *n* values used for each analysis in the figure captions.

### Study approvals.

For zebrafish study, embryos were obtained from natural spawning of WT AB zebrafish. All procedures involving zebrafish were approved by the Institutional Animal Care and Use Committee (IACUC) of Northwestern University and performed in accordance with institutional guidelines.

For the mouse study, 4-week-old male NCG mice were purchased from Charles River and castrated. Animal care and use conditions were followed in accordance with institutional and NIH protocols and guidelines, and all studies were approved by Northwestern University Animal Care and Use Committee.

### Data availability.

The next-generation sequencing data that support the findings of this study have been deposited in the Gene Expression Omnibus (GEO) under accession code of GSE296858. Values for all data points in graphs are reported in the Supporting Data Values file.

## Author contributions

YY and QC conceived and designed the research with the help of SAA and JCZ. YY performed the majority of the experiments with assistance from CY, RW, SW, Q Liu, XZ, and Q Li. XY and XH synthesized the MS-series EZH2 degraders under supervision of JJ. HZO, NX, and XD provided the IHC analysis. SN conducted the polysome profiling assay under the supervision of DA. JF performed the majority of bioinformatics analysis under the supervision of RY. YL and XG reanalyzed the MeRIP data with the supervision of KC. XZ performed the zebrafish experiment with the help of KK and EMÖ. YY wrote the paper. All authors discussed the results and commented on the manuscript. YY, JF, and CY contributed equally to this work. Authorship order was determined by the extent of each author’s experimental contributions.

## Funding support

This work is the result of NIH funding, in whole or in part, and is subject to the NIH Public Access Policy. Through acceptance of this federal funding, the NIH has been given a right to make the work publicly available in PubMed Central.

- Northwestern University startup fundings (to YY, RY, and QC).
- U.S. Department of Defense grant HT9425-23-1-0661 (to YY).
- NIH P50CA180995 SPORE in Prostate Cancer Career Enhancement Award (to YY).
- The Elsa U. Pardee Foundation Research Grant (to YY).
- NIH grants R35GM142441 and R01CA259388 (to RY).
- NIH grants R01CA256741, R01CA278832, R01CA285684, and R01CA300246 (to QC).
- Prostate SPORE P50CA180995 Development Research Program (to QC)
- The Polsky Urologic Cancer Institute of the Robert H. Lurie Comprehensive Cancer Center of Northwestern University at Northwestern Memorial Hospital (to QC).
- NIH grant R35GM159598 (to DA)
- The American Association for Cancer Research 22-20-01-ARAN (to DA).
- Canadian Institute of Health Research PJT156150 and PTJ178063 (to XD).
- Carcinogenesis Training Program T32 CA009560 (to SN).
- NIH grant R01CA268519 (to JJ).
- NIH grants R01GM125632 and R01GM138407 (to KC).
- National Cancer Institute grant P50CA180995 (to SAA).
- NIH grant R01HD103623 (to EMÖ).
- NIH grants R01CA286147 and R01CA275193 (to JCZ).

## Supplementary Material

Supplemental data

Unedited blot and gel images

Supplemental table 1

Supplemental table 2

Supplemental table 3

Supporting data values

## Version 1 11/18/2025

In-Press Preview

## Version 2 01/16/2026

Electronic publication
