# Supplementary material for: EZH2 crosstalk with RNA methylation promotes prostate cancer progression through modulation of m6A autoregulation pathway
Source: J Clin Invest. 2025 Nov 18;136(2):e195840. doi: 10.1172/JCI195840 (PMC12807473; doi:10.1172/JCI195840)
Supplement: Supplemental data [file jci-136-195840-s155.pdf]

Supplemental materials

EZH2 crosstalk with RNA methylation promotes prostate cancer progression through modulation of m^6A autoregulation pathway

Yang Yi, Joshua Fry, Chaehyun Yum, Rui Wang, Siqi Wu, Sharath Narayan, Qi Liu, Xingxing Zhang, Htoo Zarni Oo, Ning Xie, Yanqiang Li, Xinlei Gao, Xufen Yu, Xiaoping Hu, Qiaqia Li, Kemal Keseroglu, Ertuğrul M. Özbudak, Sarki A. Abdulkadir, Kaifu Chen, Jian Jin, Jonathan C. Zhao, Xuesen Dong, Daniel Arango, Rendong Yang, Qi Cao

Contents

Supplemental Figures 1-6

Supplemental Methods

Supplemental Table 4 and 5

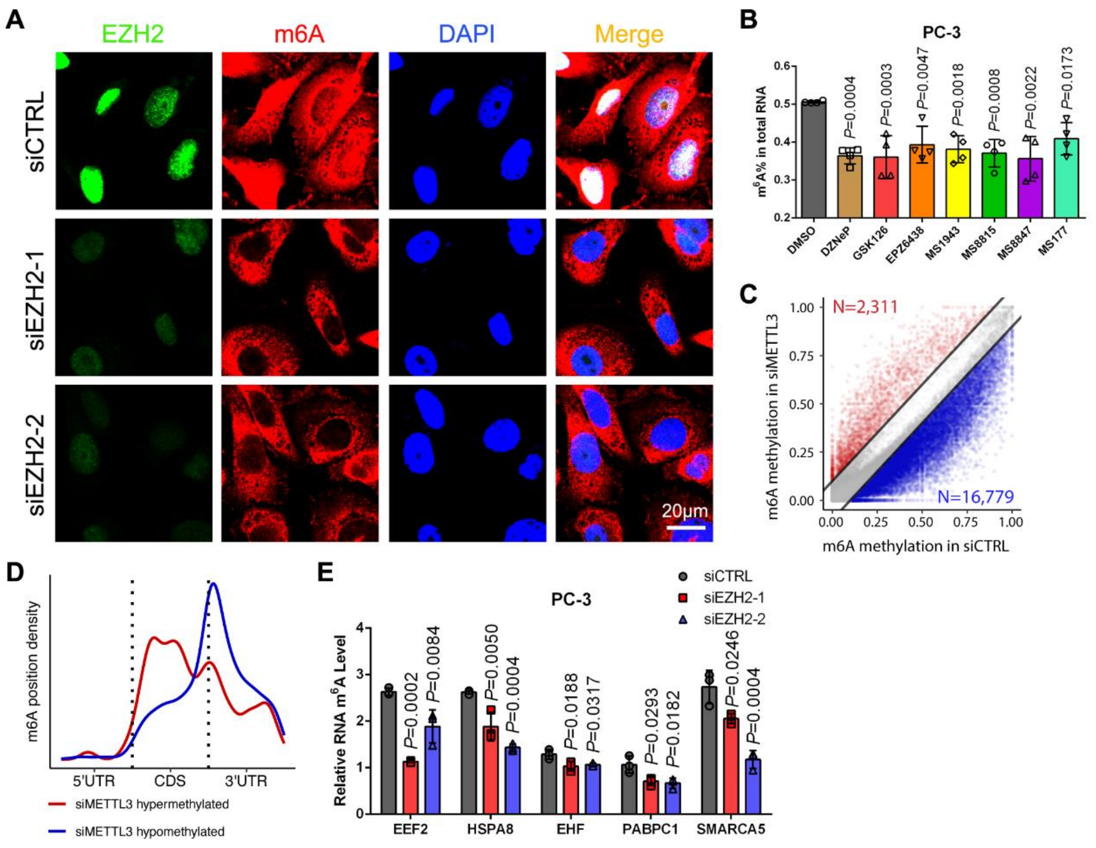

Supplemental Figure 1. EZH2 is a positive m^{6}A regulator in PCa.

(A) Representative fluorescence images to show the RNA m^{6}A staining in control and EZH2-deficient PC-3 cells. Endogenous EZH2 were co-stained by anti-EZH2 antibody and the nuclei were visualized by DAPI (Scale bar: 20 μm).

(B) The m^{6}A ELISA to measure the global m^{6}A levels in PC-3 cells treated with a series of EZH2 inhibitors. For DZNeP, GSK126, and EPZ6438, a concentration of 5 μM was used. For all the MS drugs, a concentration of 1 μM was used. Statistical significance was determined by one-way ANOVA followed by Dunnett's multiple-comparison test.

(C) Scatter plot showing the m^{6}A methylation in C4-2 cells upon METTL3 depletion, with siMETTL3 hypomethylated sites in blue and hypermethylated sites in red.

(D) The distribution of METTL3-affected m^{6}A sites across the transcript.

(E) The m^{6}A CUT&RUN-qPCR analysis to validate the EZH2-affected m^{6}A sites in each indicated transcript of PC-3 cells.

One-way ANOVA followed by Dunnett’s multiple-comparison test was used for statistical analysis in **B** and **E**.

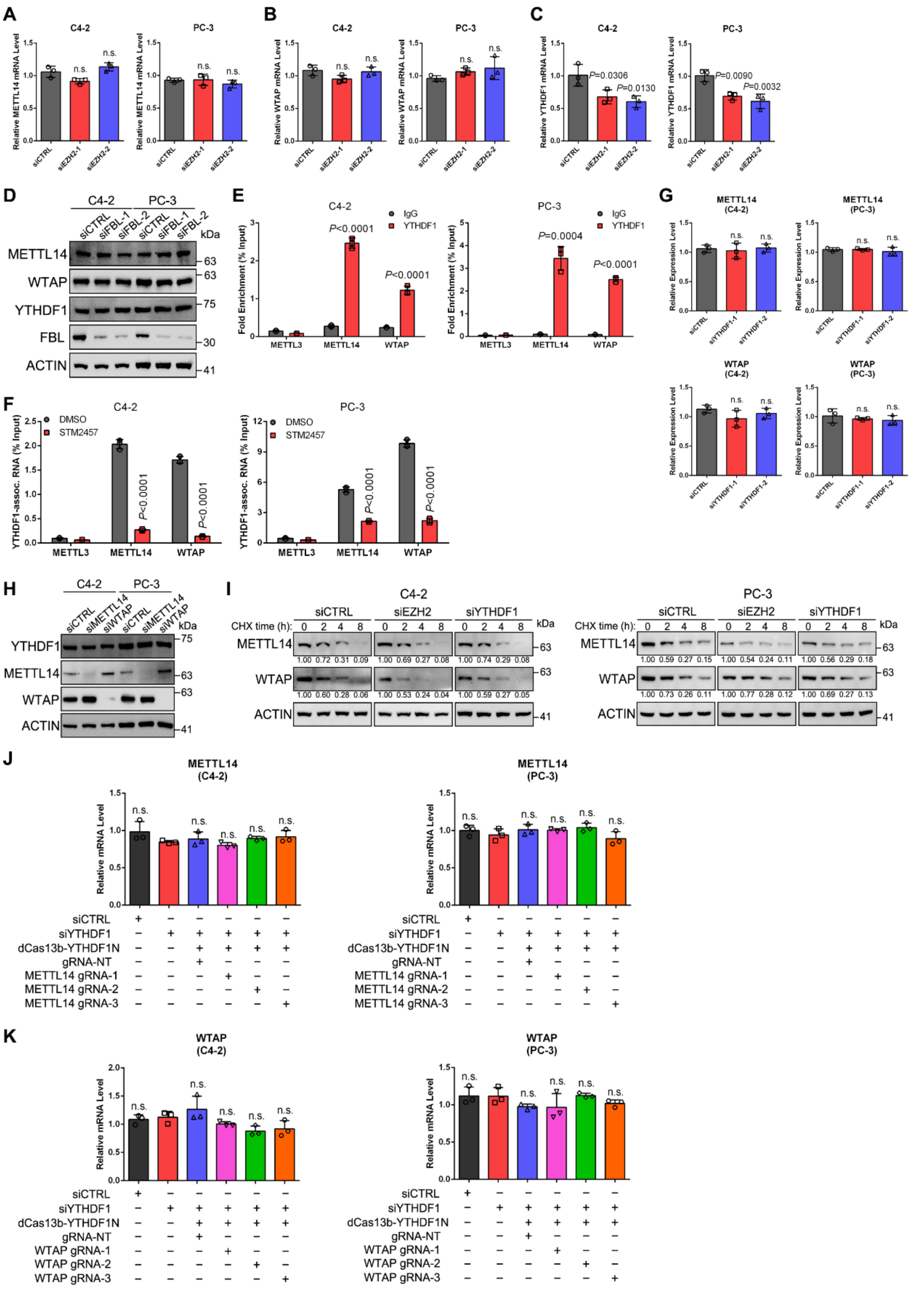

Supplemental Figure 2. EZH2 modulates an m^{6}A autoregulation pathway by targeting YTHDF1.

(A-C) RT-qPCR assay to show the mRNA changes of METTL14 **(A)**, WTAP **(B)** and YTHDF1 (**C**) upon EZH2 knockdown in two PCa cell lines.

(D) Western blot to detect the expression change of three m^6A mediators upon FBL suppression in two PC cell lines.

(E) RIP-qPCR assay in two PCa cell lines to validate the binding of YTHDF1 proteins to *METTL14* and *WTAP* mRNAs.

(F) RIP-qPCR assay in two PCa cell lines to monitor the change of YTHDF1 binding to *METTL14* and *WTAP* mRNAs upon STM2457 treatment (5 μM).

(G) RT-qPCR assay to detect the mRNA levels of *METTL14* and *WTAP* upon YTHDF1 knockdown in two PCa cell lines.

(H) Western blot to measure the expression change of YTHDF1 upon METTL14 or WTAP suppression in two PC cell lines.

(I) CHX treatment assay was performed to monitor the degradation of METTL14 and WTAP proteins in control, EZH2-deficient and YTHDF1-deficient PCa cells. The relative protein level was shown under the bands.

(J, K) YTHDF1-deficient PCa cells were transfected with dCas13b-YTHDF1N and gRNAs targeting METTL14 **(J)** or WTAP **(K)**, followed by RT-qPCR analysis to measure the change of *METTL14* and *WTAP* mRNAs, respectively.

One-way ANOVA followed by Dunnett's multiple-comparison test was used for statistical analysis in **A**, **B**, **C**, **G**, **J** and **K**. Two-tailed Student's t-test was used in **E** and **F**.

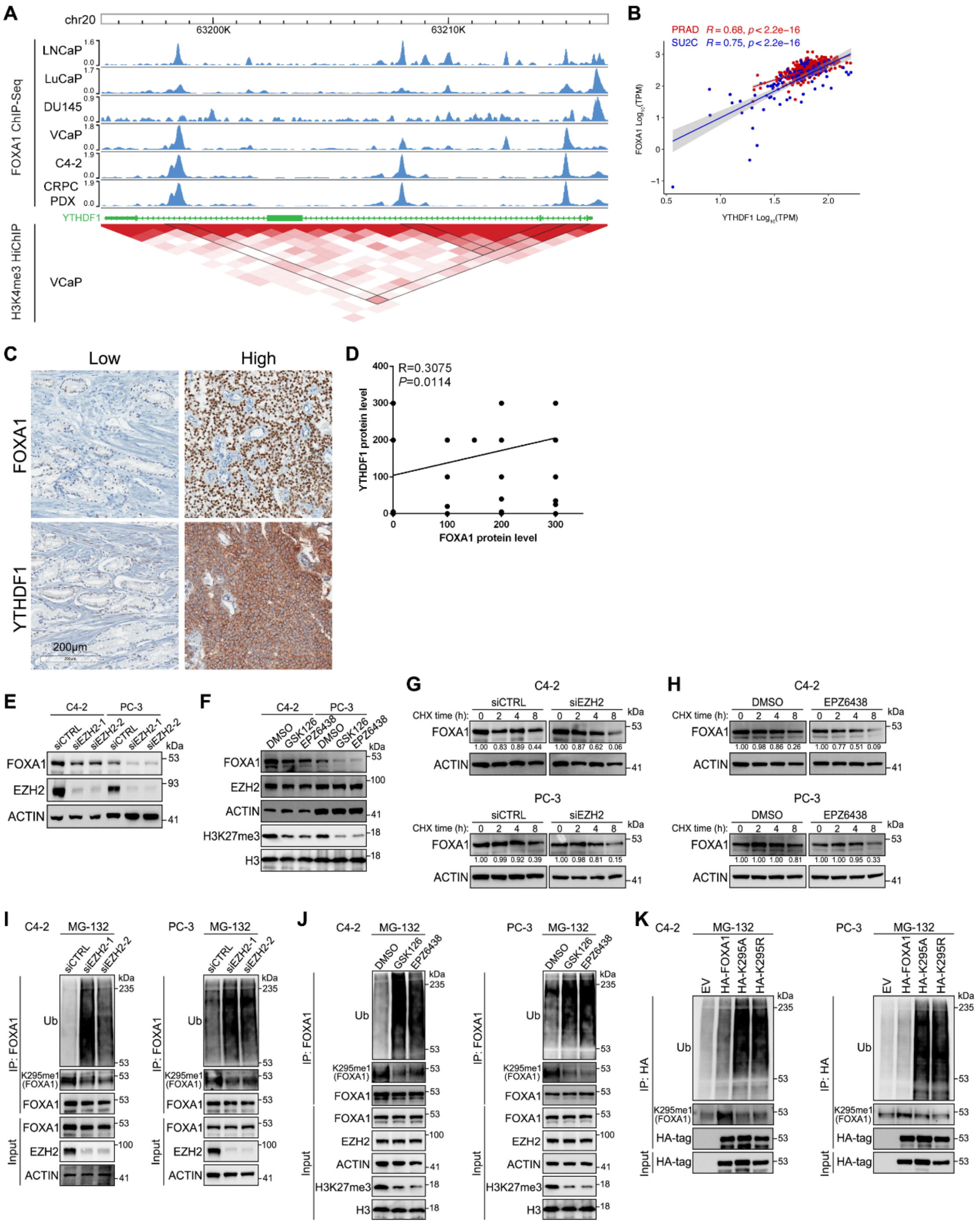

Supplemental Figure 3. EZH2 upregulates YTHDF1 transcription through FOXA1.

(A) FOXA1 binds to the promoter and distal regulatory elements of YTHDF1 in PCa cell lines and CRPC PDX model. Top data track (blue) shows ChIP-seq binding profiles along YTHDF1 locus. Bottom heatmap shows interaction strength in H3K4me3 HiChIP in VCaP cell line. Major distal interactions with promoter highlighted in black.

(B) Scatter plot showing the relationship between FOXA1 and YTHDF1 mRNA expressions using data from TCGA-PRAD and SU2C, with Spearman correlation coefficient (R) and P value as indicated. TPM: transcript per million.

(C) Representative IHC staining of PCa TMA slides using the indicated antibodies. Scale bar = 200 μm.

(D) Scatter plot showing the correlation between protein levels of FOXA1 and YTHDF1, as revealed by the PCa TMA IHC data. The R and P value were calculated as indicated.

(E) Western blot to detect the change of FOXA1 protein level upon EZH2 knockdown in two PCa cell lines.

(F) Western blot to detect the change of FOXA1 protein level upon EZH2 inhibitor treatment (5 μM for each) in two PCa cell lines.

(G, H) CHX treatment assay was performed to monitor the degradation of FOXA1 proteins upon EZH2 knockdown **(G)** or EPZ6438 treatment **(H)** in PCa cells. The relative FOXA1 protein level was shown under the bands.

(I) Co-IP of endogenous FOXA1 in control and EZH2-deficient PCa cells followed by WB analysis with the indicated antibodies. Cells were treated with 10 μM MG-132 for 14 h before subjecting to co-IP assay.

(J) Co-IP of endogenous FOXA1 in DMSO and EZH2 inhibitor-treated PCa cells followed by WB analysis with the indicated antibodies. Cells were treated with 10 μM MG-132 for 14 h before subjecting to co-IP assay.

(K) PCa cells were transfected with empty vector (EV), HA-FOXA1, HA-FOXA1-K295A or HA-FOXA1-K295R and treated with 10 μM MG-132 for 14 h before co-IP assay using anti-HA antibody.

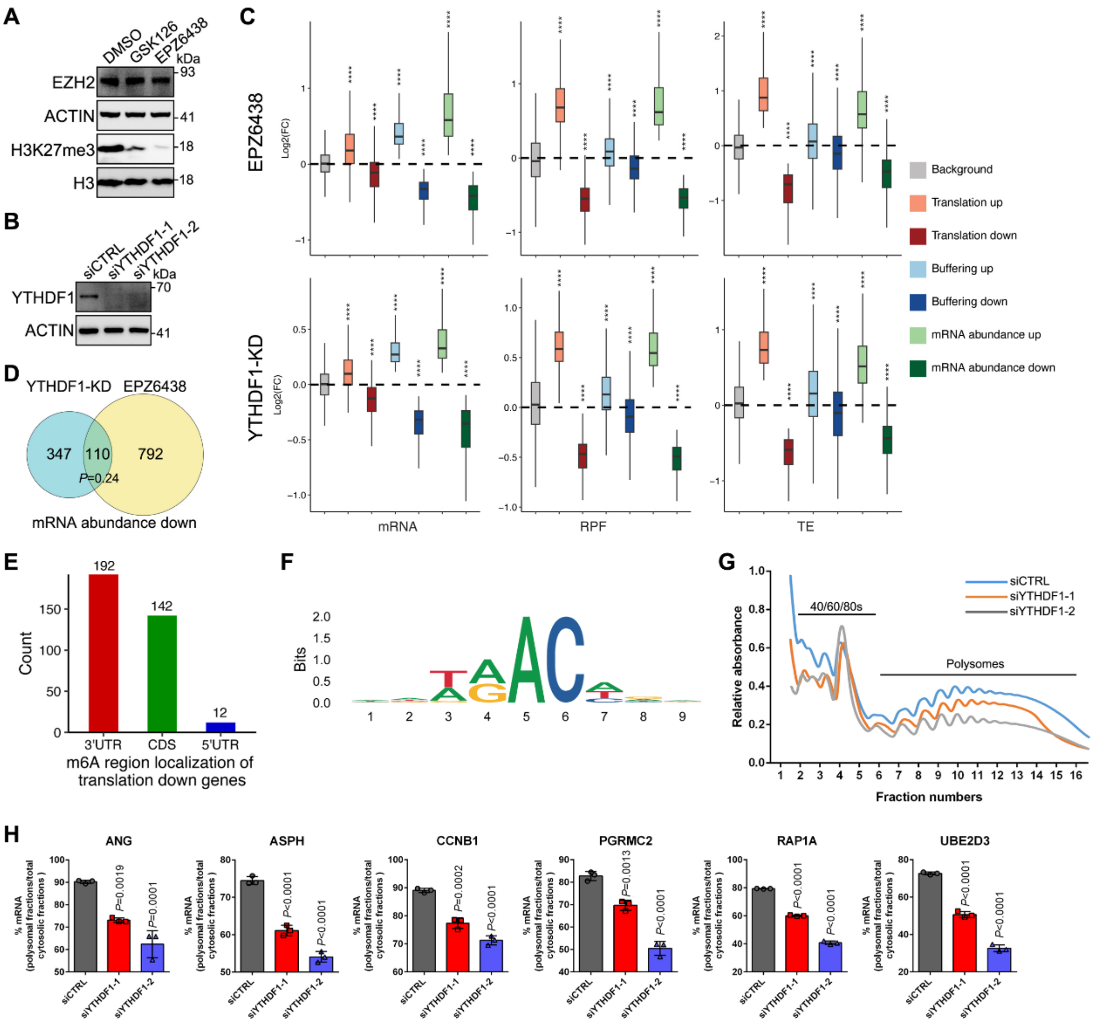

Supplemental Figure 4. EZH2 enzymatic inhibitor suppresses the translation process in PCa cells.

(A) Western blot to detect the expression of EZH2 and H3K27me3 in C4-2 cells undergoing GSK126 or EPZ6438 treatment (5 μM for each).

(B) Western blot to detect the knockdown efficiency of YTHDF1 in YTHDF1-deficient C4-2 cells.

(C) Box plots to show the log2 fold-change (log2(FC)) of the mRNA, RPF and TE in the condition of EPZ6438 treatment or YTHDF1 knockdown. Statistical significance was assessed using two-tailed Student's t-tests to determine if log2 fold-change values significantly differed from 0.

(D) Venn diagram showing the overlap between downregulated genes from mRNA

abundance mode after EPZ6438 treatment or YTHDF1 knockdown.

(E) Graph showing the m^6A peak localizations of the 70 overlapping genes identified in **Fig. 4E**.

(F) The shared m^6A sequence motif for the 70 overlapping genes identified in **Fig. 4E**.

(G) Cytoplasmic polysome patterns of control and YTHDF1-deficient C4-2 cells.

(H) Quantification of the ratio of each polysomal-bound mRNA candidate to the total cytoplasmic mRNA of its own. Statistical significance was determined by one-way ANOVA followed by Dunnett's multiple-comparison test.

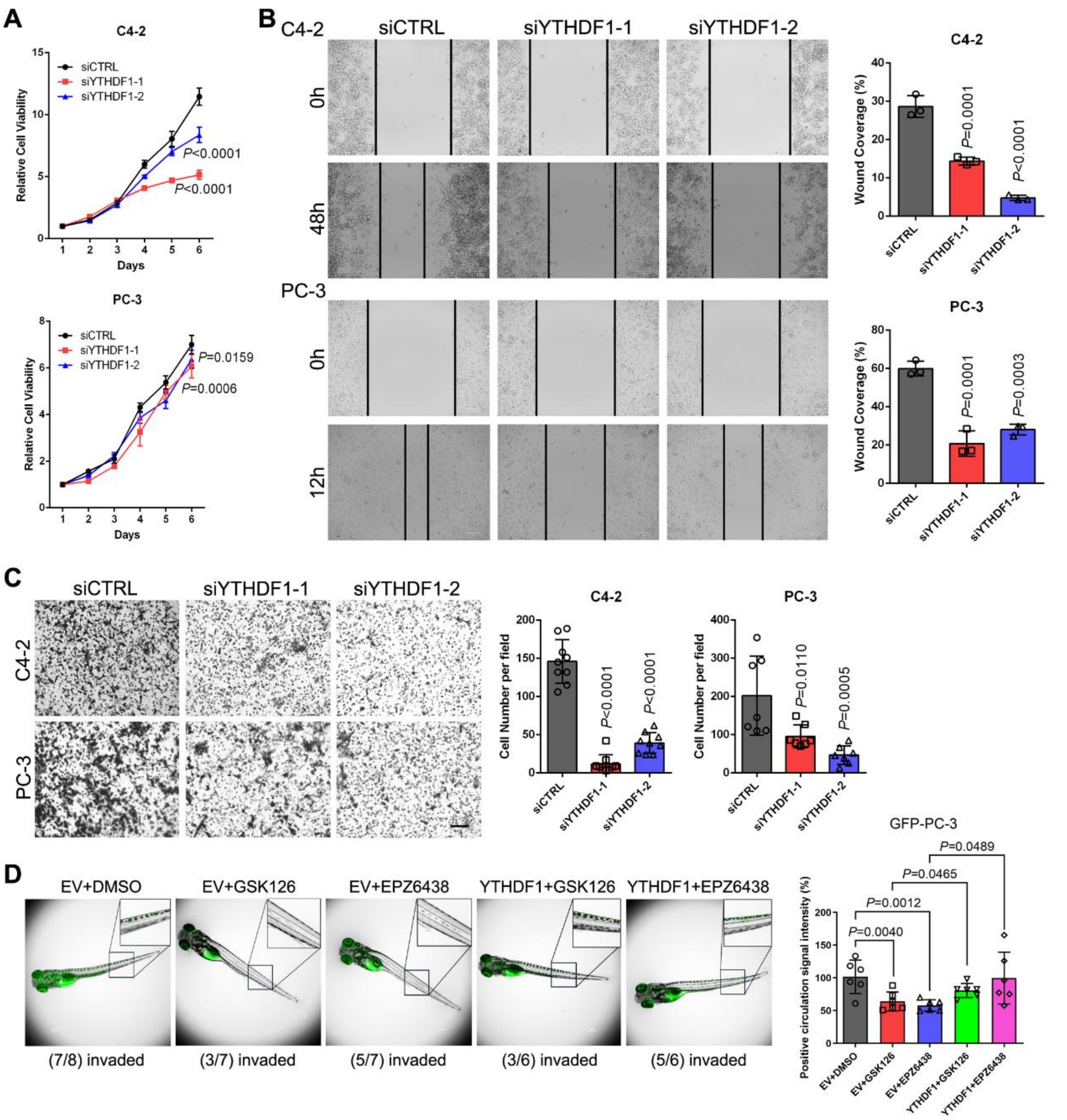

Supplemental Figure 5. YTHDF1 sustains aggressiveness of PCa cells.

(A) Cell viability assay to assess the proliferative capacity of control and YTHDF1-deficient PCa cells.

(B) Wound healing assay to evaluate the migration potential of PCa cells after YTHDF1 depletion. The healing of wounded cell layer was monitored under a microscope at the indicated time-points. Graph showing the rate of filling of the scratched area by cells.

(C) Boyden chamber invasion assay to determine the invasive capability of PCa cells after YTHDF1 depletion. Graph showing the number of migrated cells passing through Matrigel at 24 h.

(D) GFP-labeled PC-3 cells in each condition were injected into zebrafish embryos. Tumor cell invasion was examined upon 3 days and images were taken under 4 × magnification. Embryos exhibiting positive circulation signals were classified as “invaded”. Graph showing the mean fluorescence intensity (%), with individual data points representing each measurement. For each group, three zebrafish larvae were randomly selected, and two distinct regions per larvae were analyzed to measure fluorescence intensity.

One-way ANOVA followed by Dunnett’s multiple-comparisons test was used for statistical analysis in **A**, **B**, **C** and **D**. For comparisons involving only two groups in **D**, an unpaired two-tailed Student’s t-test was applied.

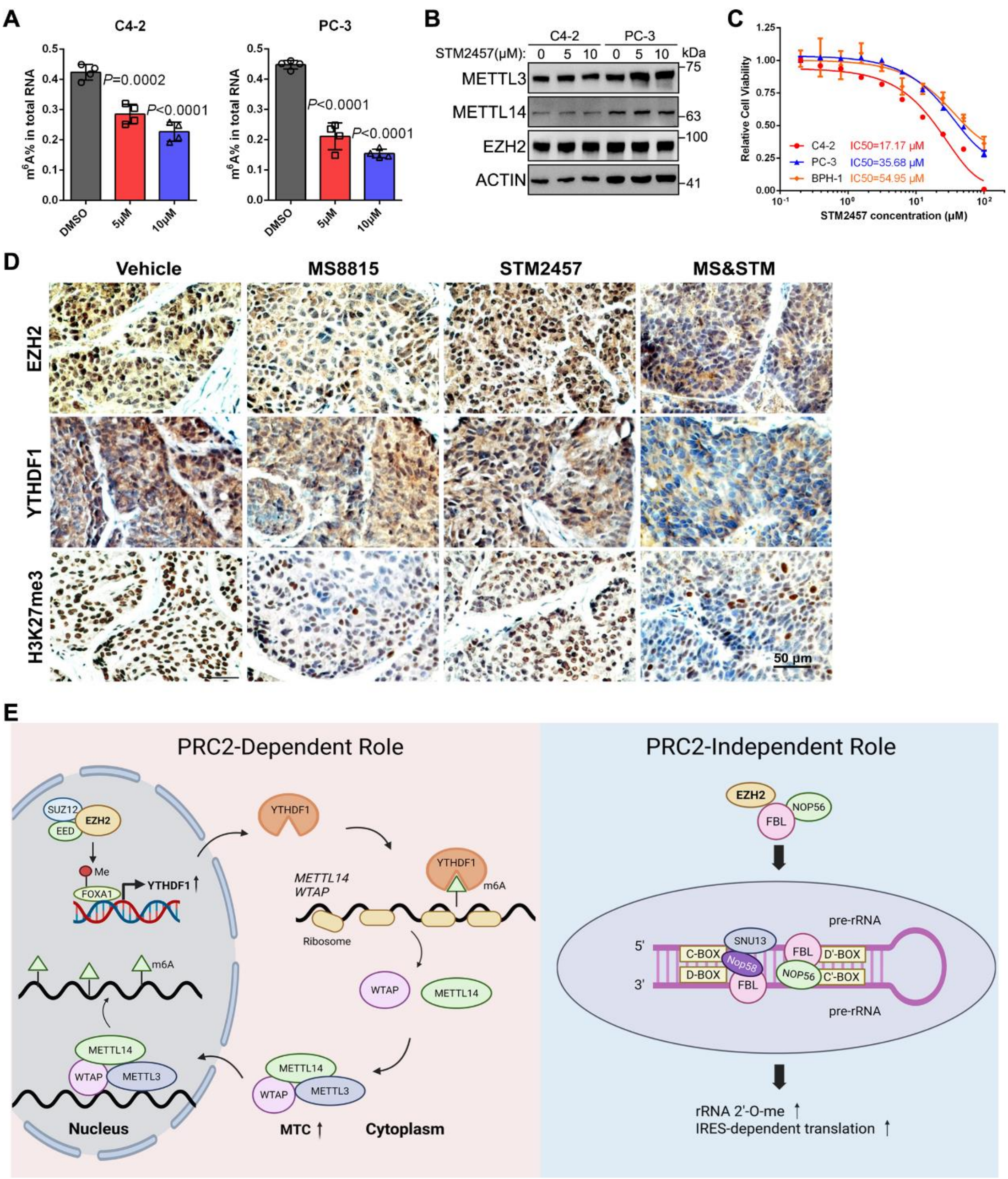

Supplemental Figure 6. MS8815 and STM2457 synergize in PCa therapy.

(A) The m^6A ELISA to measure the m^6A changes in two PCa cell lines treated with different STM2457 concentrations as indicated. Statistical analysis was determined by one-way ANOVA followed by Dunnett's multiple-comparisons.

(B) Western blot to detect the expression of indicated proteins in each group of **A**.

(C) The IC_{50} curves of STM2457 in three prostate cell lines as indicated.

(D) Representative IHC staining images of the PDX tumor slides using the indicated

antibodies. Scale bar: 50 μm.

(E) Model illustrating the role of EZH2 in cancer-related translational control. On one hand, EZH2 globally enhances mRNA translation by promoting FBL function through a PRC2-independent mechanism. On the other hand, EZH2 specifically increases the translation efficiency of a subset of m^6A-marked transcripts via activating the EZH2-m^6A signaling pathway, which relies on its lysine methyltransferase activity. Created with BioRender.com.

Supplemental Methods

Western blot

Cell or tissue lysates were mixed with 2 × loading buffer (Bio-Rad) and heated at 95 °C for 10 min to denature proteins. Protein samples were then subjected to standard SDS-PAGE and semi-dry transferred to PVDF membranes (Bio-Rad). After blocking for 45 min in Tris-buffered saline-Tween 20 (TBST) with 5% nonfat milk, membranes were incubated with primary antibodies for 2 h at room temperature. Subsequently, membranes were washed three times using TBST and incubated with goat anti-mouse/rabbit IgG (H+L)-HRP secondary antibody (GenDEPOT, 1:5000 dilution) for 1 h. The signals were developed using western ECL substrate (Bio-Rad) and visualized by a Bio-Rad imaging system. The primary antibodies used for WB in this paper were listed in **Supplemental Table 4**.

RT-qPCR analysis

The total RNA was isolated using RNeasy Plus Mini Kit (Qiagen), followed by reverse transcription into cDNA using Maxima H Minus First Strand cDNA Synthesis Kit (Thermo Fisher). The cDNA samples were amplified using Universal SYBR Green Supermix (Bio-Rad) in a QuantStudio 6 Flex Real-time PCR System (GE Healthcare) following manufacturer's instructions. All RT-qPCR primers used here were summarized in **Supplemental Table 5**. The relative transcription level was calculated using the $2^{-\Delta\Delta Ct}$ method with the Ct values normalized to GAPDH.

RIP-qPCR analysis

The RIP-qPCR experiment was performed in triplicates using the EZ-Magna RIP kit (Millipore) by following the procedure provided by the manufacturer. In brief, cells were lysed using RIP lysis buffer and incubated with antibody-magnetic beads mixture at 4 °C overnight to enrich YTHDF1 proteins along with the associated mRNAs. These

interacting mRNAs were eluted by Proteinase K digestion, followed by purification and reverse transcription into cDNA. Then, RT-qPCR assay was performed to measure the %Input of YTHDF1-binding mRNAs in each group using the primers presented in **Supplemental Table 5**.

ChIP-qPCR analysis

The ChIP experiment was performed using the EZ-Magna ChIP kit (Millipore) with the manual provided by the manufacturer. Cells were cross-linked using paraformaldehyde solution (Invitrogen) and terminated by glycine solution. Chromatin fragment at an average size of 200 bp was obtained by sonication using a Diagenode bioruptor. DNA was isolated from samples by incubation with the antibody at 4 °C overnight followed by washing and reversal of cross-linking. To analyze the enrichment of DNA fragments, qPCR assay was conducted using the primers listed in **Supplemental Table 5**. Immunoprecipitated DNA was calculated as percentage of input DNA.

Immunofluorescence

PCa cells were cultured on Millicell EZ slides (Sigma) for 24 h before immunofluorescence staining. After fixation in 4% paraformaldehyde for 15 min, cells were permeabilized in PBS containing 0.5% Triton X-100 for 10 min at room temperature. Slides were rinsed thrice with PBS and blocked with 5% BSA in PBS for 45 min. Then, the slides were co-incubated with mouse anti-EZH2 antibody and rabbit anti-m^{6}A antibody at 4 ºC overnight. After wash with PBS, the slides were exposed to Alexa Fluor 488 conjugated goat anti-mouse and Alexa Fluor 555 conjugated goat anti-rabbit antibodies (1:200, Invitrogen) for one hour at 37 ºC, followed by three washes with PBS. Cell nuclei were stained in the dark with DAPI (Invitrogen) and mounted using ProLong Diamond Antifade Mountant (Invitrogen). Immunostained cells were

viewed and photographed using Nikon A1R confocal microscope. The RNase inhibitor was added throughout the experiment to avoid RNA degradation. The primary antibody information was listed in **Supplemental Table 4**.

MG-132 and CHX treatment assays

To determine the change of nascent protein synthesis efficacy, PCa cells were cultured in 6-well plate and treated with MG-132 (Sigma) at a concentration of 10 μM for 8 h. For both treated and untreated groups, cell lysates were harvested and subjected to immunoblotting to detect the protein levels of METTL14 and WTAP.

To measure the protein degradation rate, PCa cells were cultured in 6-well plate and exposed to CHX (Sigma) at a concentration of 25 μg/mL. Cell lysates were harvested at the indicated time-points after CHX addition, followed by immunoblotting to detect the protein levels of METTL14 and WTAP at each time-point. The percentage of protein remaining at the indicate points was quantified by densitometry after normalization with β-actin. The amount of detected protein at the zero time in each group was considered as 1.

Co-IP assay

For ubiquitination detection, transfected or drug-treated cells were treated with 10 μM MG-132 for 14 hours before subjected to co-IP. The whole-cell lysate was made by lysing cells in NP-40 lysis buffer (Thermo) for 15 min, followed by sonication at 5 s on and 5 s off for 1 min. Insoluble material was then separated by centrifugation. Lysates were pre-incubated with Dynabeads protein A/G (Invitrogen) to eliminate nonspecific binding. Then, antibodies were mixed into the lysates with newly added Dynabeads and incubated at room temperature for 2 h. The immune complexes were collected using a magnetic separator and washed three times with NP-40 buffer. Bound proteins were eluted with 2× reducing SDS sample buffer (Bio-Rad) for 10 min at 95 °C before WB

analysis. All primary antibodies used for co-IP were listed in **Supplemental Table 4**.

Tissue microarrays (TMAs) and Immunohistochemistry (IHC) staining

Prostate tumor biopsies retrieved from Vancouver Prostate Centre tissue bank were recruited to construct TMAs as described previously (1). This protocol was approved by the office of research ethics in the University of British Columbia. IHC was performed using Ventana Discovery XT autostainer (Ventana) with the indicated antibodies by following its manual. All stained slides were recorded by a Leica SCN400 scanner. Digital images were evaluated and scored by a pathologist, Dr. Htoo Zarni Oo. The histology score (H-score) of each stained protein was calculated by the Aperio ImageScope software (Leica Biosystems) based on both intensity and percentage of the IHC signals.

For IHC in PDX, harvested tumors were fixed in 4% paraformaldehyde solution for 48 hours. After fixation, the tissue was embedded into paraffin wax. The paraffin-embedded tissue block was then sliced into 8 μm sections using a microtome, and then mounted on Polysine microscope adhesion slides (Epredia) for staining. Histological tissue slides were routinely deparaffinized in Xylene and rehydrated through a series of alcohol treatments. Antigen retrieval treatment was performed by protease-induced epitope retrieval followed with heat-induced epitope retrieval. Tissues were pre-treated by trypsin solution (0.25%) and incubated for 20 minutes at 37 °C in humidified chamber. Then allowed sections to cool down to room temperature and rinse the sections in distilled water before soaking them in 100 ml working solution of Citrate-Based Antigen Unmasking Solution (VectorLabs). After treatment, the endogenous peroxidase activity was blocked with 3% H_2O_2 with raise buffer for 10 min. After blocking in 1% goat serum in IHC Select Immunoperoxidase Secondary Detection System (Millipore), immunohistochemical blotting was performed using the indicated

antibodies. Omitting the primary antibodies was served as the negative control. Biotinylated secondary goat anti-mouse IgG/goat anti-rabbit IgG antibody (Millipore) was used in labelling with IHC Select Immunoperoxidase Secondary Detection System (Millipore). VectaMount Express mounting medium (VectorLabs) was used to preserve the histochemical stains. Then slides were examined and photographed using a Nikon Scope Upright with an Olympus Camera. All primary antibodies used for IHC were listed in **Supplemental Table 4**.

Cell viability assay

CellTiter-Glo 2.0 Luminescent Cell Viability Assay (Promega) was performed to detect the cell viability. In brief, cells were seeded in 96-well plates at a density of 5×10^3 cells/well, and were incubated under 37 °C in a humidified 5% CO_2 atmosphere. At each time-point, culture medium was discarded and 50 μL of CellTiter-Glo 2.0 solution was added into each well, followed by incubation for 10 min on an orbital shaker at 37 °C to induce cell lysis. The bioluminescence was detected using a Tecan plate reader.

For IC_{50} measurement, PCa cells were seeded in 96-well plates and treated at drug concentration gradients for 72 h. Bioluminescence was measured to quantify cell viability using CellTiter-Glo 2.0 Luminescent Cell Viability Assay (Promega) and was read on a Tecan plate reader. The cell proliferation curve was drawn and fit by the bioluminescence to drug concentration. Half-maximum inhibitory concentration (IC_{50}) was calculated with non-linear fitting.

Wound healing assay

PCa cells were cultured in 35 mm dish with 3-well Culture-Insert (Ibidi) till grown to 90% confluency. After removing the inserts gently, dishes were refilled with serum-free RPMI 1640 medium and maintained in a humidified atmosphere at 37 ºC. Pictures were taken under microscope at the indicated time-points and the distance of cell

migration was measured by ImageJ software.

Boyden chamber invasion assay

Matrigel-coated Transwell inserts (Millipore) were used for this assay. The upper surface of the Transwell chambers was coated with Matrigel matrix (Corning) diluted in cell medium at a dilution of 1: 20. Around 1×10^4 Cells diluted in 300 μL of serum-free medium were seeded into the upper compartments of the chambers. Meanwhile, the lower compartments of the chambers were filled with 800 μL of medium with 10% FBS. After 24 h, invasive cells that had migrated from Matrigel to the lower surface of the chamber were fixed in methanol, stained with 0.1% crystal violet (Sigma) and subjected to microscopic inspection. The number of invasive cells was calculated as the average number of cells counted in 9 random fields per filter.

Colony formation assay

PCa cells expressing empty vector or ectopic YTHDF1 were seeded into six-well plates at a ratio of 8,000 cells per well. The seeded colonies were treated with 5 μM DMSO, GSK126 or EPZ6438 for one week. At the end-point, cells were fixed with 4% paraformaldehyde for 15 min at room temperature, followed by staining with 0.025% crystal violet for 1 hour. The ImageJ software was used to calculate colony area percentages.

Supplemental Table 4. Antibodies used in this study.

Antibody Target	Source	Application
EZH2	Cell signaling, Cat#5246	WB, IHC
	BD, Cat#612666	IF
m^6A	Cell signaling, Cat #56593	IF
METTL3	Cell signaling, Cat#86132	WB
METTL14	Cell signaling, Cat#48699	WB
WTAP	Cell signaling, Cat#41934	WB
VIRMA	Cell signaling, Cat#88358	WB
FTO	Cell signaling, Cat#45980	WB
ALKBH5	Cell signaling, Cat#80283	WB
YTHDF1	Cell signaling, Cat#57530	WB, RIP, IHC, IF
YTHDF2	Cell signaling, Cat#71283	WB
YTHDF3	Cell signaling, Cat#80303	WB
YTHDC1	Cell signaling, Cat#77422	WB
YTHDC2	Cell signaling, Cat#46324	WB
Histone H3	Cell signaling, Cat#4499	WB
β-Actin	Cell signaling, Cat#3700	WB
H3K27me3	Cell signaling, Cat#9733	WB, IHC
Flag-tag	Cell signaling, Cat#14793	WB
HA-tag	Cell signaling, Cat# 3724	WB, co-IP
FOXA1	Cell signaling, Cat#53528	WB, ChIP, co-IP
	Cell signaling, Cat#59197	IF
FOXA1-	A gift from Dr. Jindan Yu	WB

K295me1	(Emory University) (2)	
Ubiquitin	Cell signaling, Cat#20326	WB
Puromycin	Millipore, Cat#MABE343	WB
FBL	Abcam, Cat#ab5821	WB
CCNB1	Cell signaling, Cat#12231	WB
RAP1A	Cell signaling, Cat#2399	WB

Supplemental Table 5. Oligonucleotides used for this study.

Name	Sequence (5´to 3´)	Application
GAPDH	F: GGAGCGAGATCCCTCCAAAAT	RT-qPCR,
	R: GGCTGTTGTCATACTTCTCATGG	RIP-qPCR
METTL3	F: TTGTCTCCAACCTTCCGTAGT	RIP-qPCR
	R: CCAGATCAGAGAGGTGGTGTAG	
METTL14	F: AGTGCCGACAGCATTGGTG	RT-qPCR,
	R: GGAGCAGAGGTATCATAGGAAGC	RIP-qPCR
WTAP	F: CTTCCCAAGAAGGTTCGATTGA	RT-qPCR,
	R: TCAGACTCTCTTAGGCCAGTTAC	RIP-qPCR
YTHDF1	F: ACCTGTCCAGCTATTACCCG	RT-qPCR
	R: TGGTGAGGTATGGAATCGGAG	
YTHDF1	F: CGAACAAAGCCCGCAGG	ChIP-qPCR
	R: CAAGGTCCGAAGACTAAACGC	
ANG	F: CTGGGCGTTTTGTTGTTGGTC	RT-qPCR,
	R: GGTTTGGCATCATAGTGCTGG	RIP-qPCR
ASPH	F: CATGGAGGACACAAGAATGGG	RT-qPCR,
	R: CCAAACGACAGCTACAGATGT	RIP-qPCR
CCNB1	F: AATAAGGCGAAGATCAACATGGC	RT-qPCR,
	R: TTTGTTACCAATGTCCCCAAGAG	RIP-qPCR
PGRMC2	F: CTCTGCCTCGCATGAAGAAG	RT-qPCR,
	R: CTTTGGTCACGTCGAAGACTTT	RIP-qPCR
RAP1A	F: CGTGAGTACAAGCTAGTGGTCC	RT-qPCR,
	R: CCAGGATTTCGAGCATACACTG	RIP-qPCR

UBE2D3	F: CAGGTCCAGTTGGGGATGATA<br>R: CCGCCTTGATATGGGCTGTC	RT-qPCR, RIP-qPCR
EEF2	F: CAGGCGTTCCCCCAGTGT<br>R: ACAGTCTCCAGGTGTCGTCTGAG	m^6A CUT&RUN
HSPA8	F: GGAGGAATGCCTGGGGGA<br>R: CTGCCACAGAATTTGCTACGAA	m^6A CUT&RUN
EHF	F: GGAATAAGAGCACAACGGCAC<br>R: GCAACAGAATCCTCATCCAACAT	m^6A CUT&RUN
PABPC1	F: AAACTTTGAACCTTATGTACCGAGC<br>R: TGACATTCTGAGCTATTCCACAGTAA	m^6A CUT&RUN
SMARCA5	F: CGGGAGAATGGGATGTATGTG<br>R: TTCTGAAGAGGTGTTCCAGTTAATAATA	m^6A CUT&RUN
METTL3	F: AAACAGTGTAAAAGGAGGGGCA<br>R: GCATATTTGATATGATGATAGGCAGTC	m^6A CUT&RUN
METTL14	F: ACCAAAATCGCCTCCTCCC<br>R: CACGGCCAGCAGAAGTTCC	m^6A CUT&RUN
WTAP	F: AGGAGACACGCCAGCAGTTG<br>R: CCATTACTTGGTCCGTTTGTCAG	m^6A CUT&RUN
ANG	F: TTTCCGTCGTCCGTAACCAG<br>R: AACAGAAAACAAAAGGTCCAGGTAG	m^6A CUT&RUN
ASPH	F: TATGTGTGTTTTTCCGACAGTGG<br>R: TACACAAGTCAGTCCAACAGTTAGTGT	m^6A CUT&RUN
CCNB1	F: GCAGCACCTGGCTAAGAATGT<br>R: GCTTCGATGTGGCATACTTGTT	m^6A CUT&RUN

PGRMC2	F: TGTAAACAACCAAAGTCAGGGGC	m^{6}A CUT&RUN
	R: TTCGCAGCAGGTGAATCAAAC	
RAP1A	F: TCCCTGGAGAAAAAAATTGCTC	m^{6}A CUT&RUN
	R: AAATGCATCCATGCTCTTTTGTA	
UBE2D3	F: TCAGCAGAACAGAAAATGTGATGTA	m^{6}A CUT&RUN
	R: TTAACTAATAGCTCCAAGCCTCCTAA	
METTL14 gRNA-1	Oligo-1: CACCCATTAGCATGAATGAAGTCCCCG	dPspCas13b assay
	Oligo-2: CAACCGGGGACTTCATTCATGCTAATG	
METTL14 gRNA-2	Oligo-1: CACCCTTCCAAATAGATGAAGGCGTCT	dPspCas13b assay
	Oligo-2: CAACAGACGCCTTCATCTATTTGGAAG	
METTL14 gRNA-3	Oligo-1: CACCAGTCAAGTAGGAATTAGGAGCAC	dPspCas13b assay
	Oligo-2: CAACGTGCTCCTAATTCCTACTTGACT	
WTAP gRNA-1	Oligo-1: CACCGTACATTTACACTTGAGTCCAAG	dPspCas13b assay
	Oligo-2: CAACCTTGGACTCAAGTGTAAATGTAC	
WTAP gRNA-2	Oligo-1: CACCGTCTACACTTTCATACCCCGCAC	dPspCas13b assay
	Oligo-2: CAACGTGCGGGGTATGAAAGTGTAGAC	
WTAP gRNA-3	Oligo-1: CACCCCCTGTGAAATCCAGACCCAGAC	dPspCas13b assay
	Oligo-2: CAACGTCTGGGTCTGGATTTCACAGGG	

References

1. Yu, Y., Yang, O., Fazli, L., Rennie, P.S., Gleave, M.E., and Dong, X. 2015. Progesterone receptor expression during prostate cancer progression suggests a role of this receptor in stromal cell

differentiation. *Prostate* 75:1043-1050.
2. Park, S.H., Fong, K.W., Kim, J., Wang, F., Lu, X., Lee, Y., Brea, L.T., Wadosky, K., Guo, C., Abdulkadir, S.A., et al. 2021. Posttranslational regulation of FOXA1 by Polycomb and BUB3/USP7 deubiquitin complex in prostate cancer. *Sci Adv* 7.
