# Supplementary material for: EZH2 crosstalk with RNA methylation promotes prostate cancer progression through modulation of m6A autoregulation pathway
Source: J Clin Invest. 2025 Nov 18;136(2):e195840. doi: 10.1172/JCI195840 (PMC12807473; doi:10.1172/JCI195840)

Fig.1B

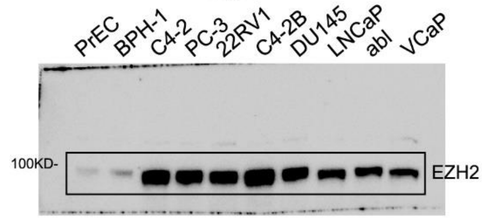

Fig.1B

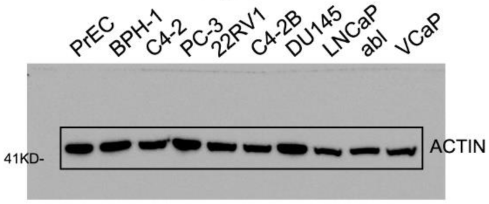

Fig.2A

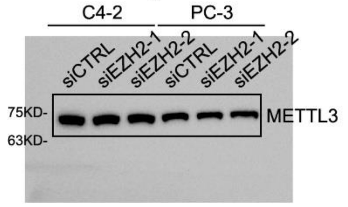

Fig.2A

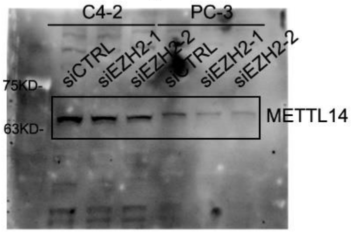

Fig.2A

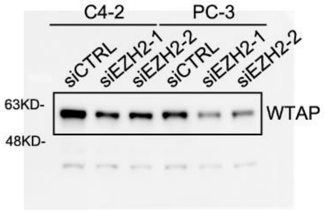

Fig.2A

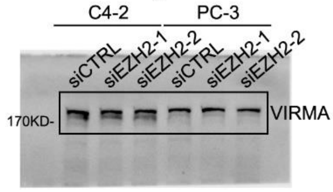

Fig.2A

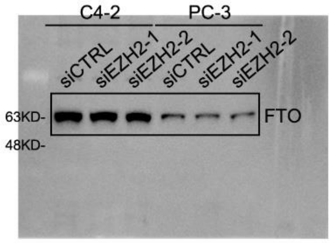

Fig.2A

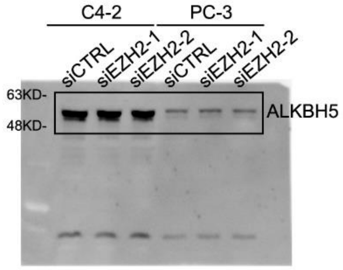

Fig.2A

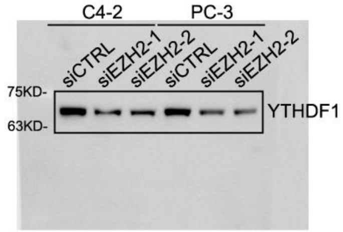

Fig.2A

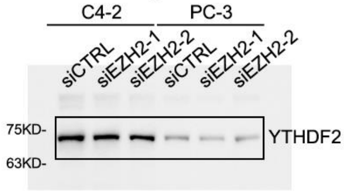

Fig.2A

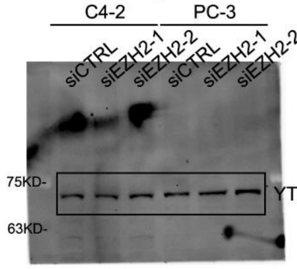

Fig.2A

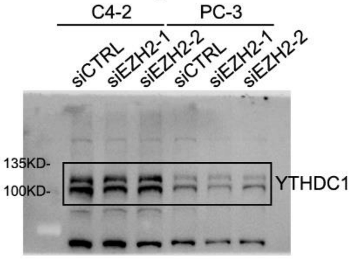

Fig.2A

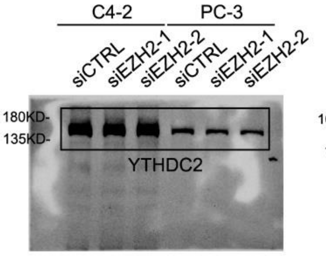

Fig.2A

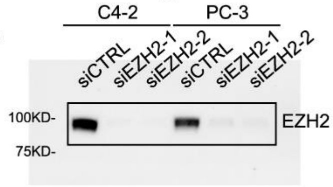

Fig.2A

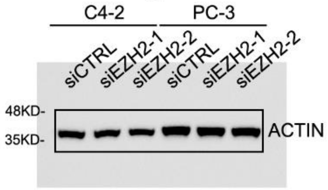

Fig.2A

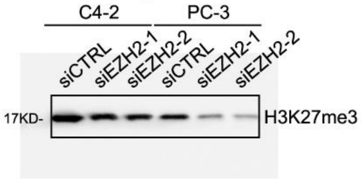

Fig.2A

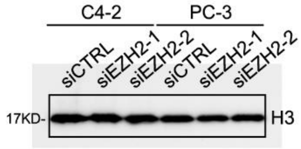

Fig.2F

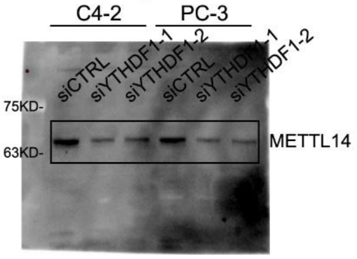

Fig.2F

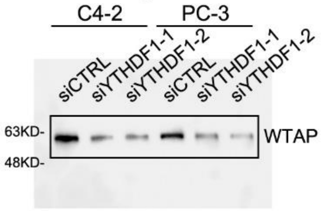

Fig.2F

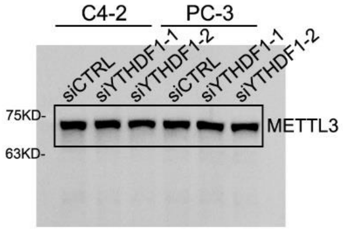

Fig.2F

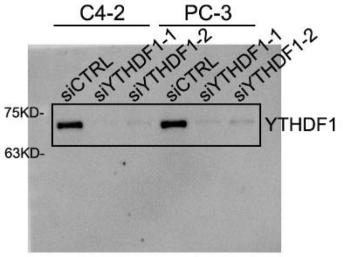

Fig.2F

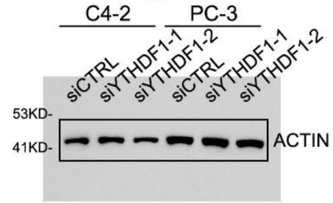

Fig.2G

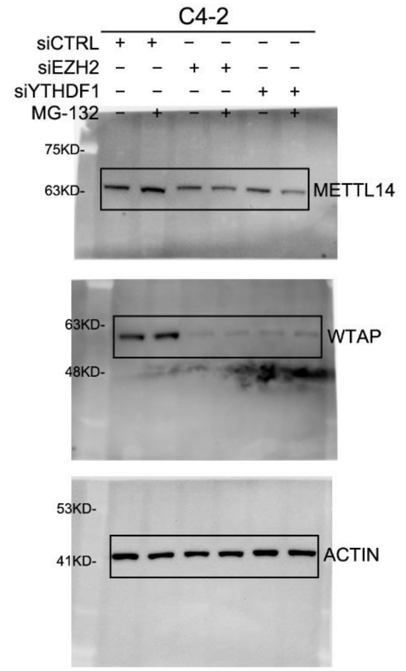

Fig.2H

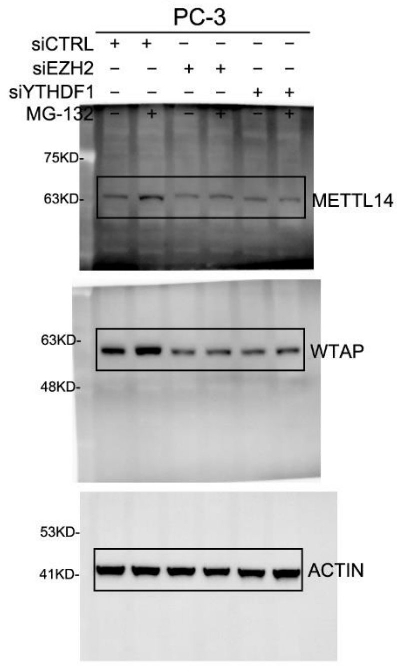

Fig.2J

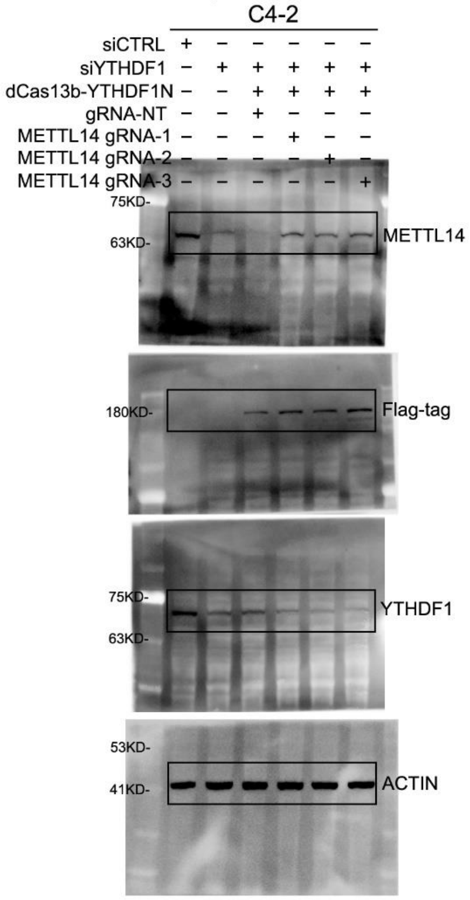

Fig.2J

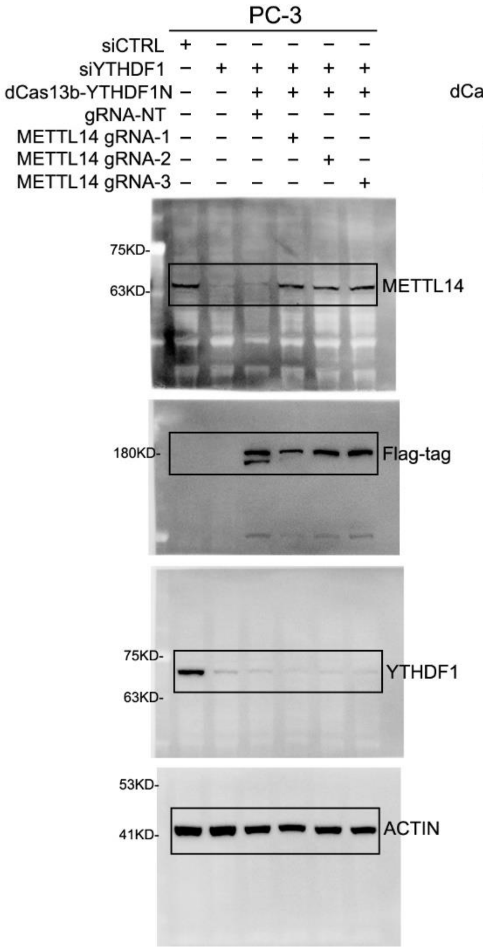

Fig.2K

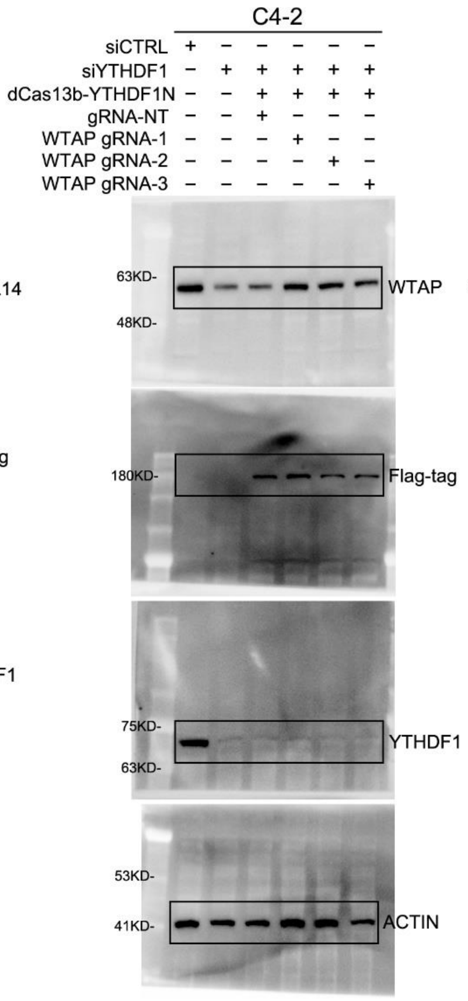

Fig.2K

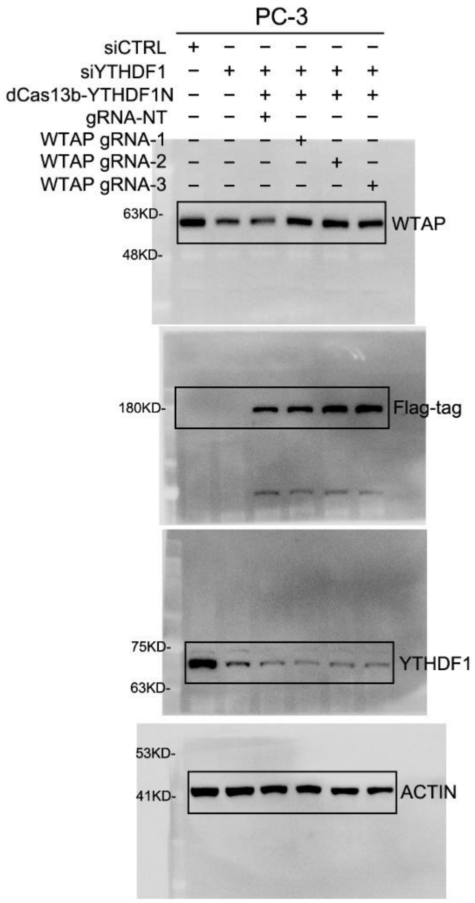

Fig.S2D

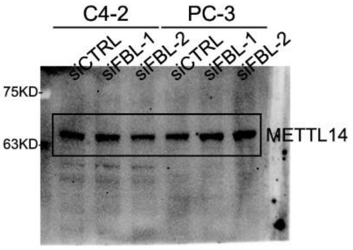

Fig.S2D

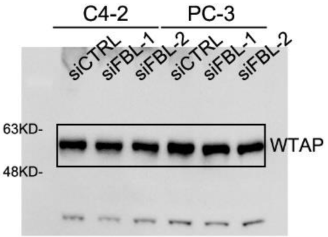

Fig.S2D

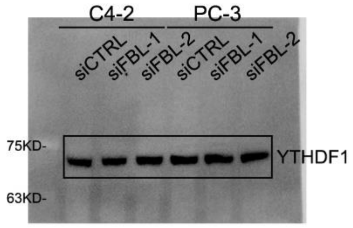

Fig.S2D

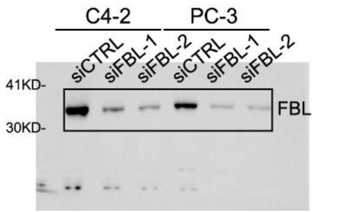

Fig.S2D

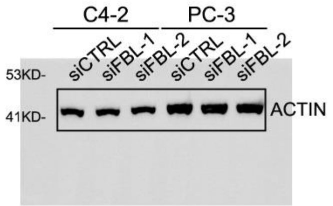

Fig.S2H

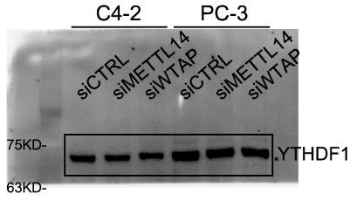

Fig.S2H

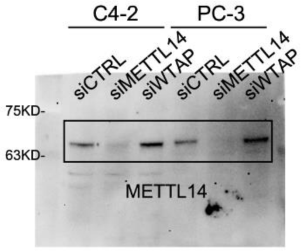

Fig.S2H

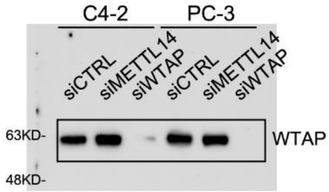

Fig.S2H

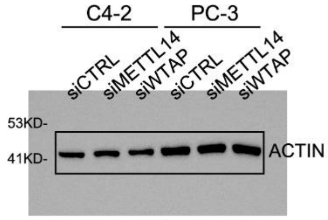

Fig.S2I

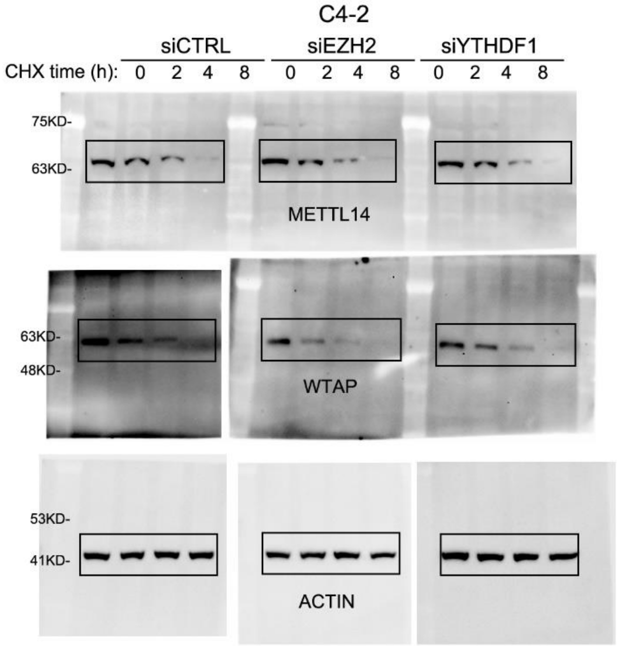

Fig.S2I

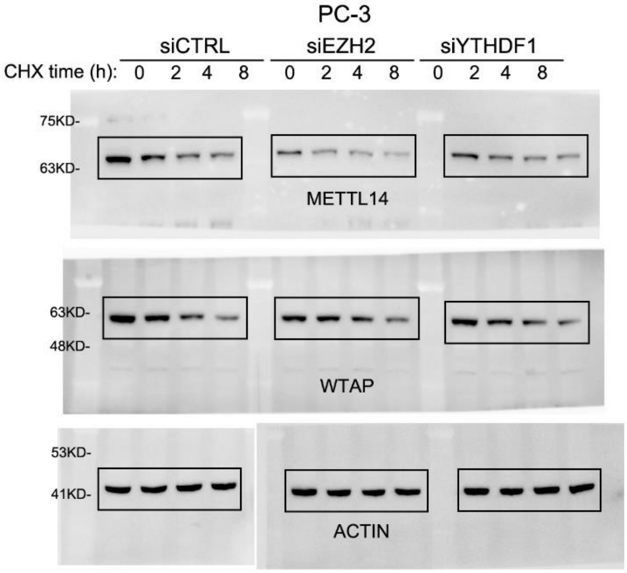

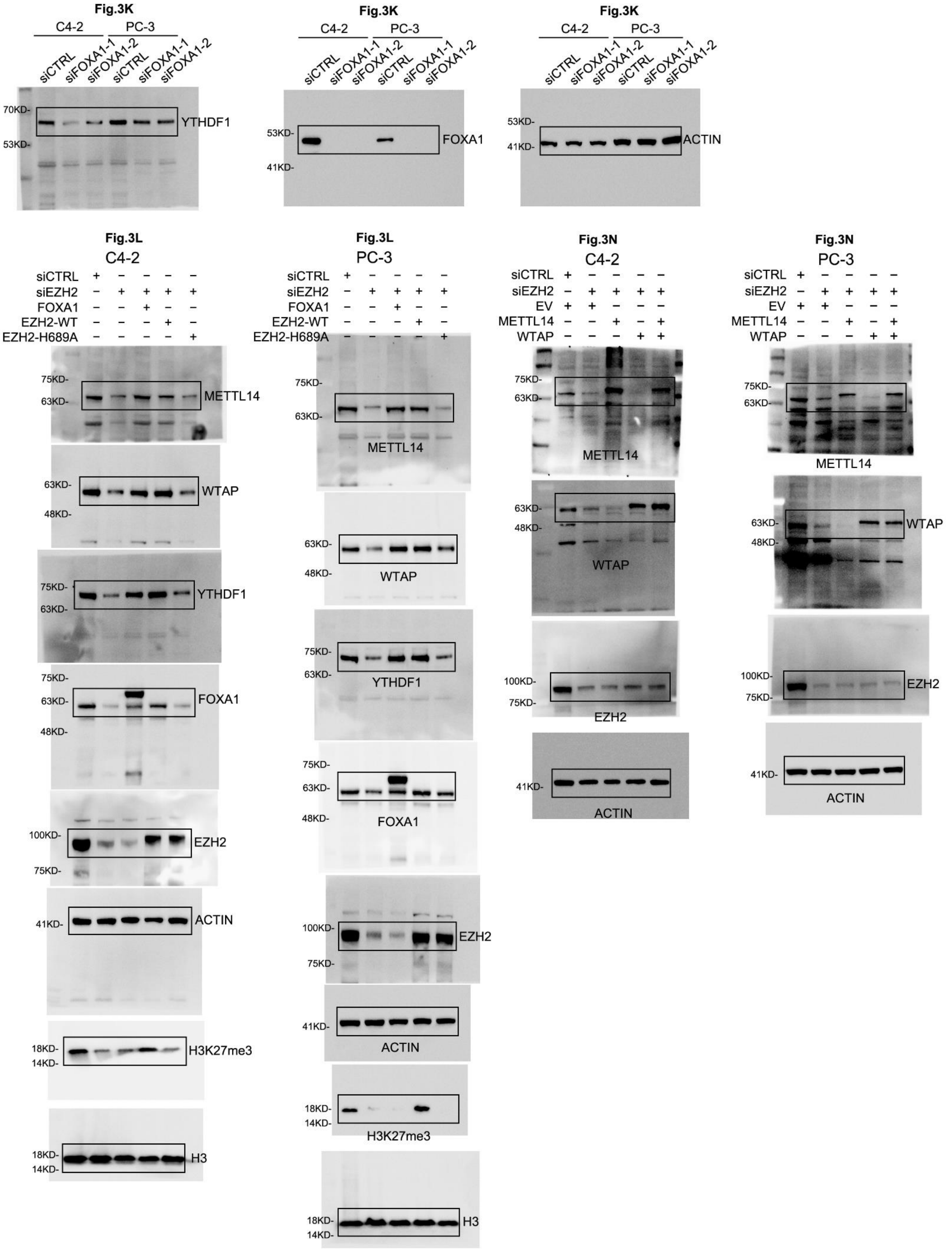
Fig.3K
C4-2
PC-3
siCTRL
siFOXA1-1
siFOXA1-2
70KD-
53KD-
YTHDF1
41KD-
FOXA1
ACTIN
Fig.3L
C4-2
PC-3
siCTRL
siEZH2
FOXA1
EZH2-WT
EZH2-H689A
75KD-
63KD-
48KD-
100KD-
18KD-
14KD-
METTL14
WTAP
EZH2
H3K27me3
H3
Fig.3N
EV

Fig.S3E

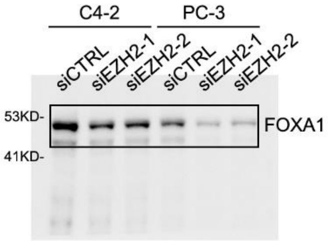

Fig.S3E

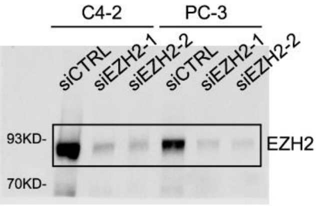

Fig.S3E

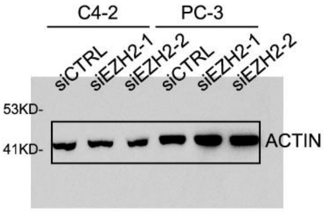

Fig.S3F

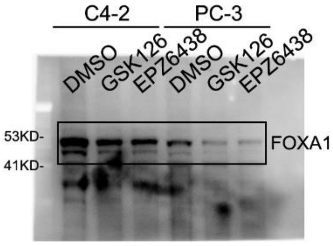

Fig.S3F

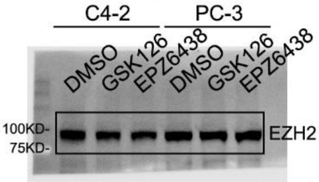

Fig.S3F

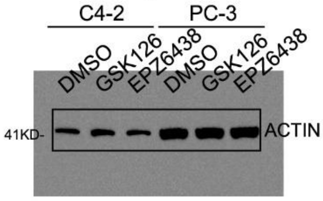

Fig.S3F

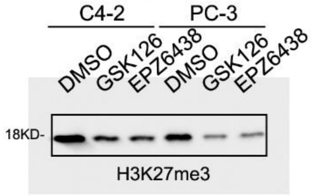

Fig.S3F

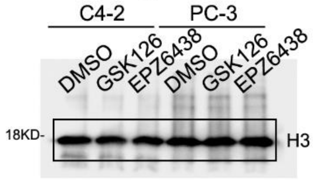

Fig.S3G

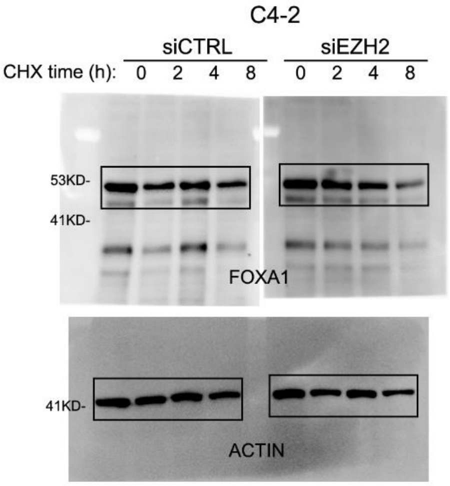

Fig.S3G

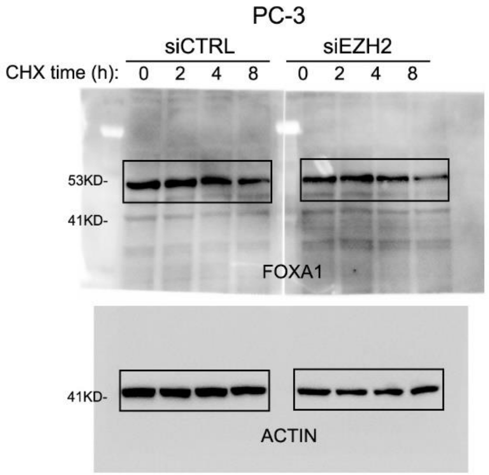

Fig.S3I

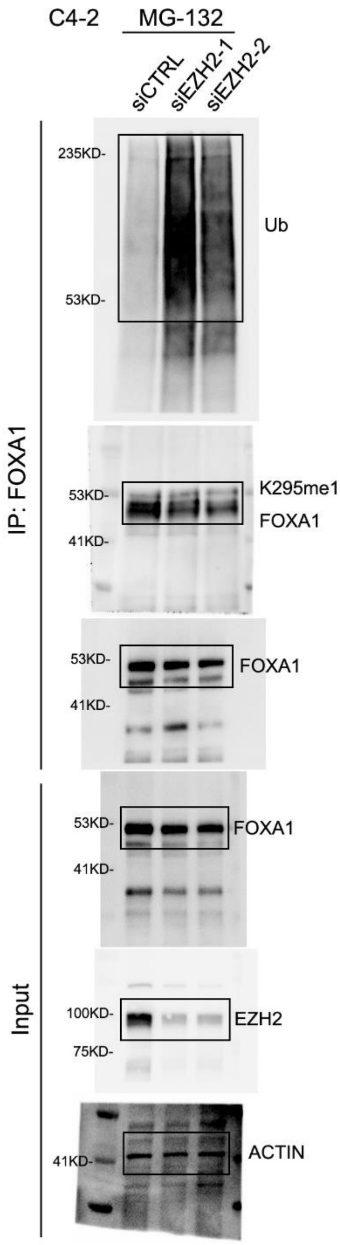

Fig.S3H

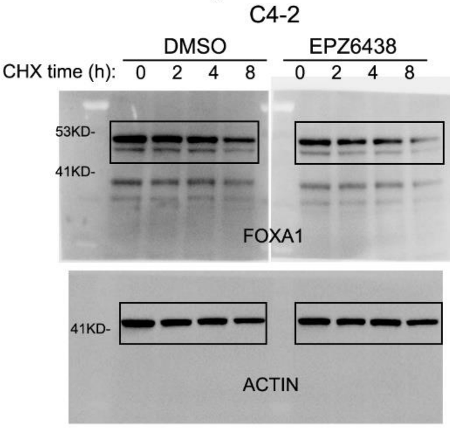

Fig.S3H

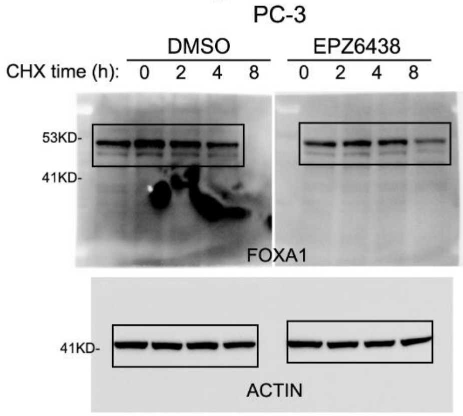

Fig.S3I

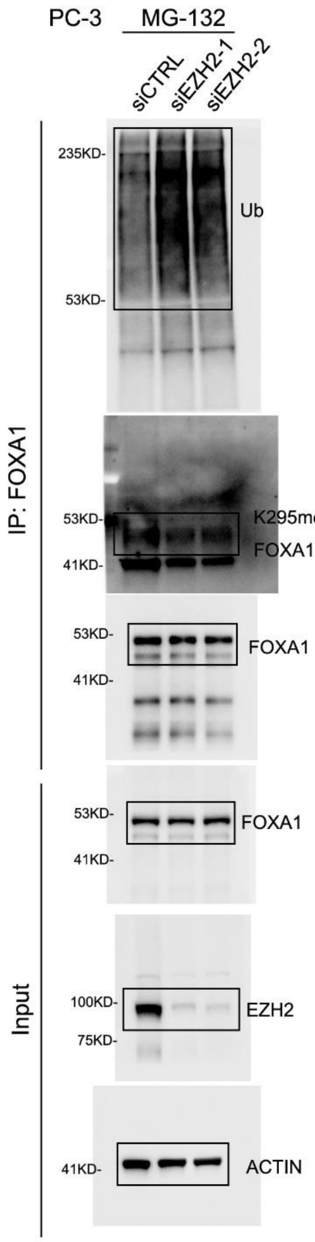

Fig.S3J

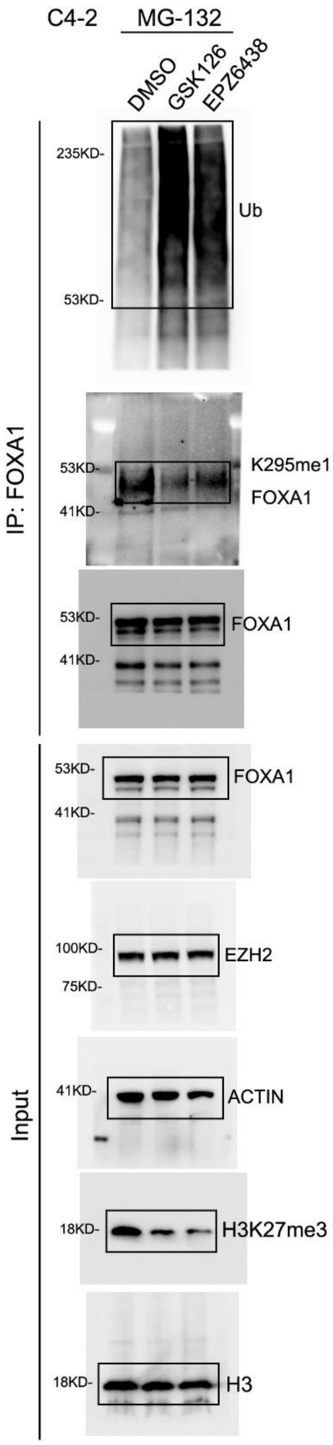

Fig.S3J

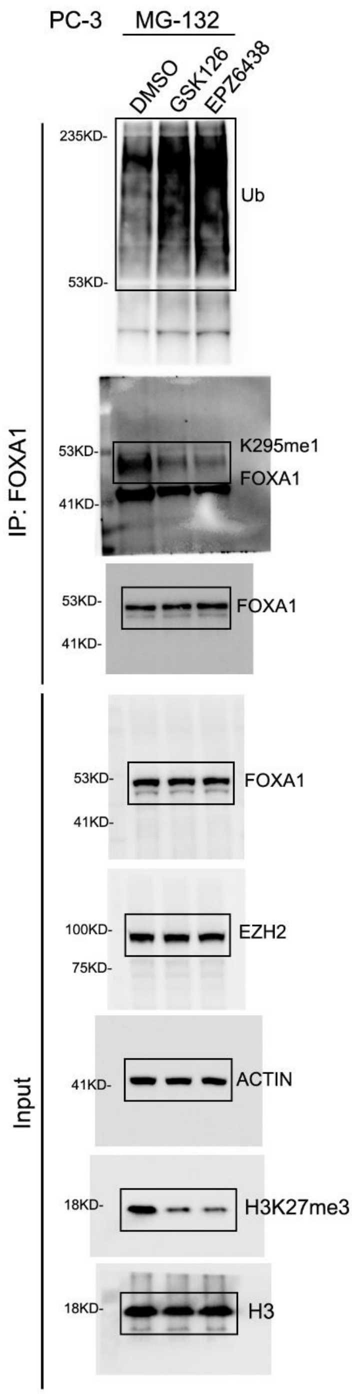

Fig.S3K

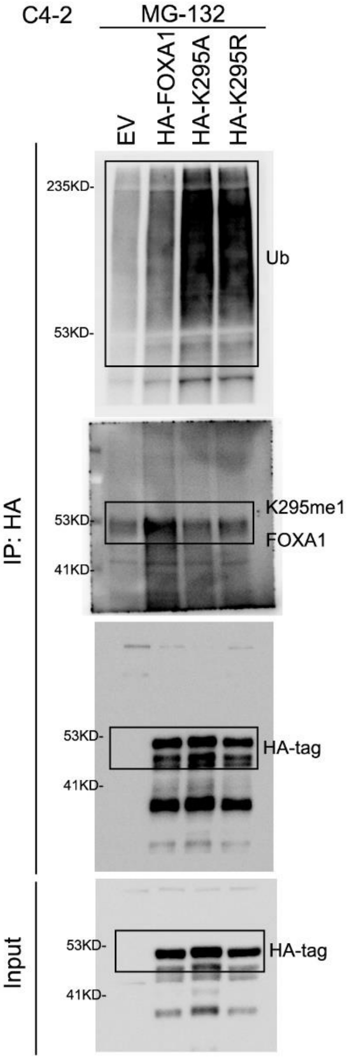

Fig.S3K

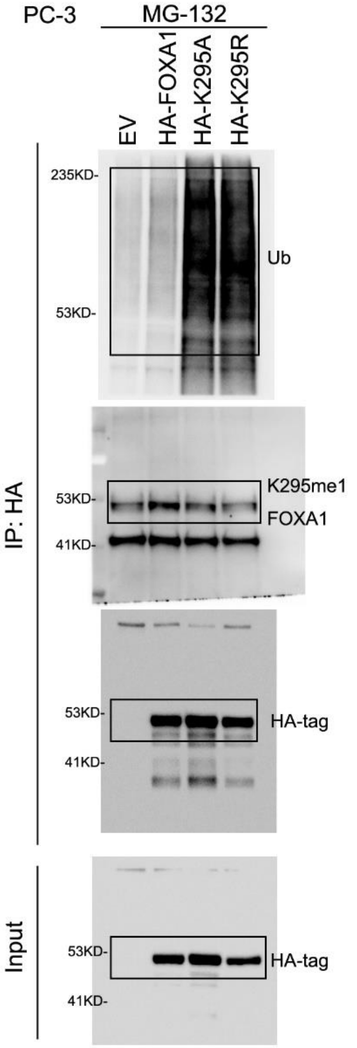

Fig.4A

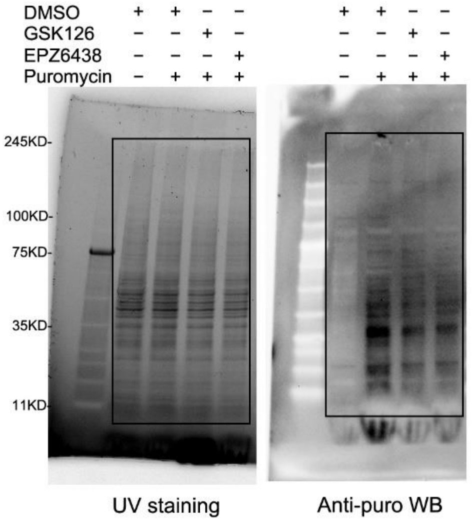

Fig.4B

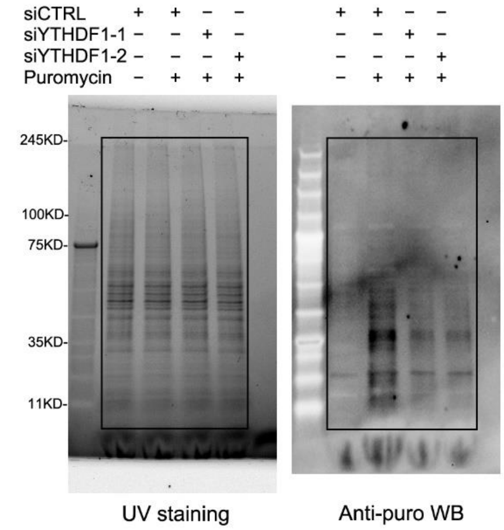

Fig.S4A

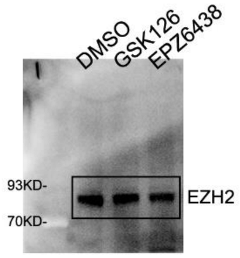

Fig.S4A

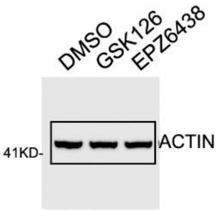

Fig.S4A

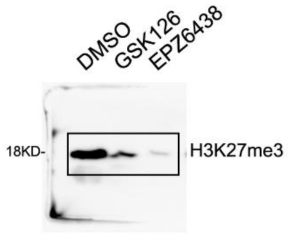

Fig.S4A

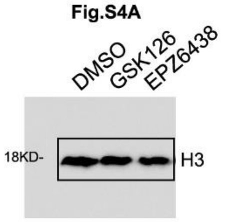

Fig.S4B

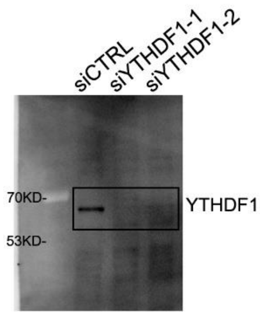

Fig.S4B

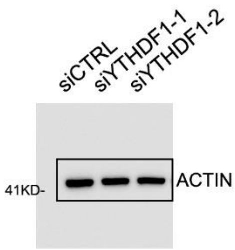

Fig.5C

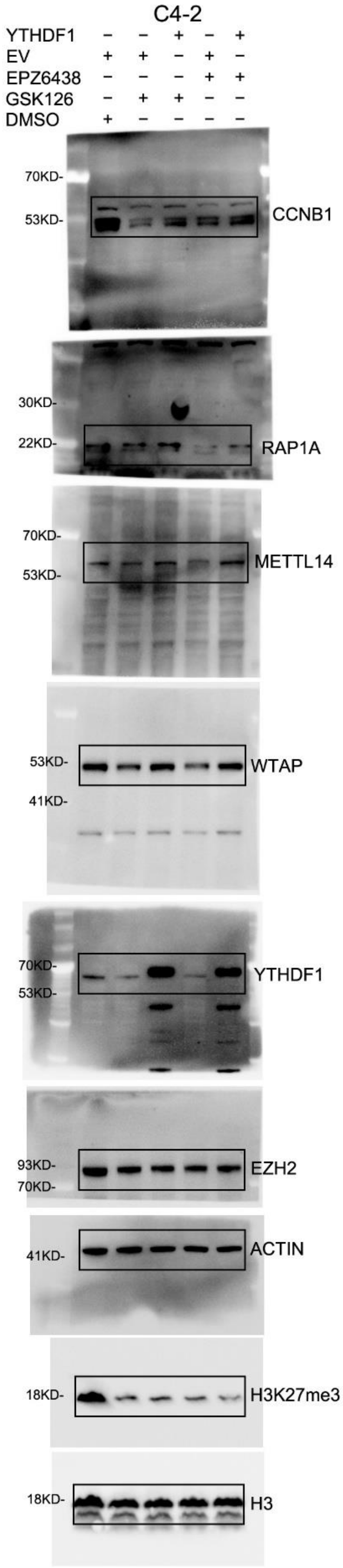

Fig.5C

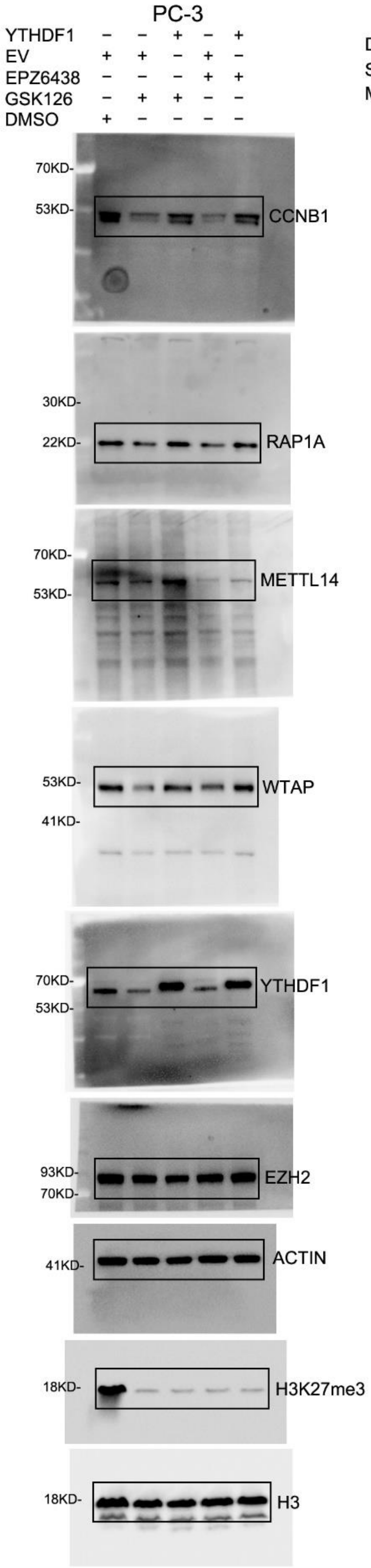

Fig.6B Fig.6B

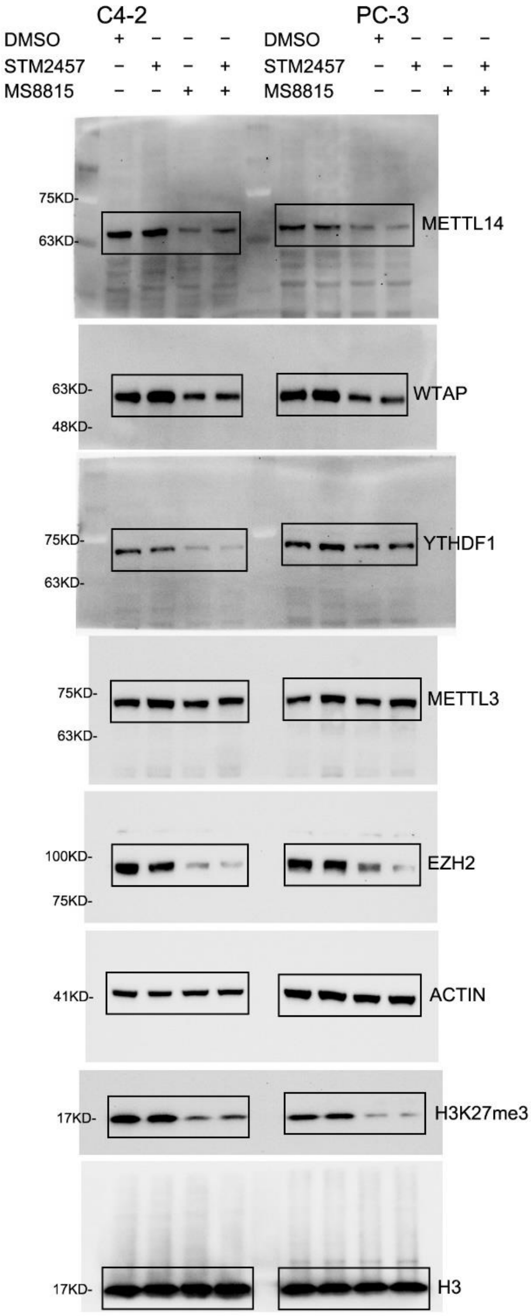

Fig.6G

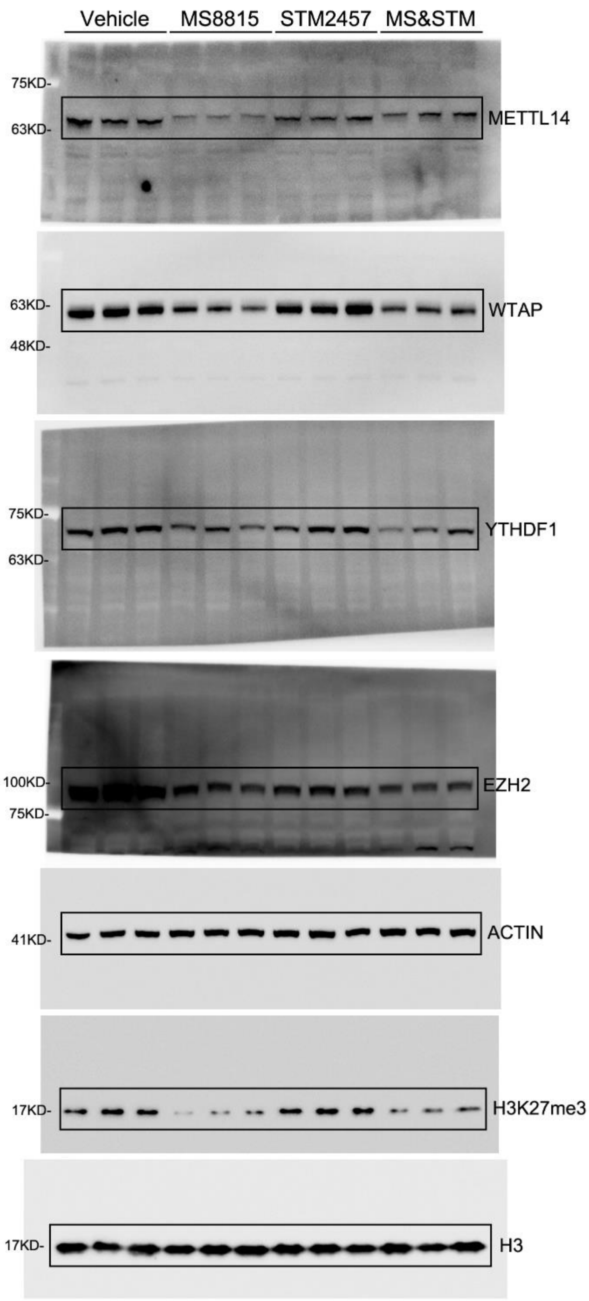

Fig.S6B

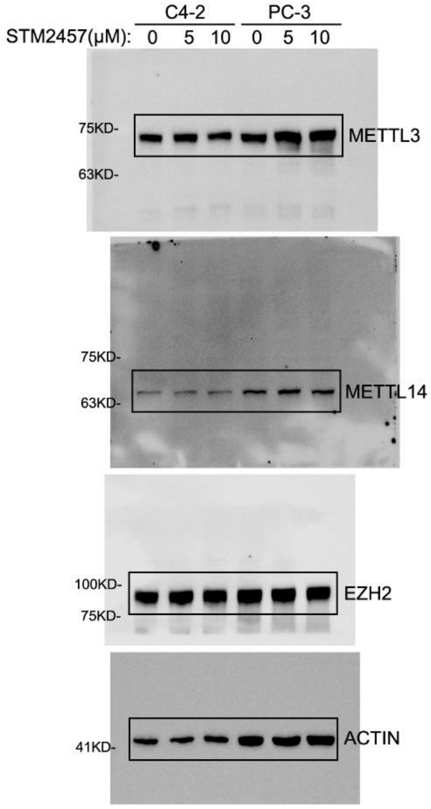

Supplement: Unedited blot and gel images [file jci-136-195840-s156.pdf]
